# Rsr1 Palmitoylation and GTPase Activity Status Differentially Coordinate Nuclear, Septin, and Vacuole Dynamics in Candida albicans

**DOI:** 10.1128/mBio.01666-20

**Published:** 2020-10-13

**Authors:** T. Bedekovic, E. Agnew, A. C. Brand

**Affiliations:** a Medical Research Council Centre for Medical Mycology at the University of Exeter, Exeter, United Kingdom; b School of Medicine, Medical Sciences and Nutrition, University of Aberdeen, Aberdeen, United Kingdom; Karlsruhe Institute of Technology (KIT)

**Keywords:** *Candida albicans*, GTPase signaling, cell polarity, hyphal development, nuclear division, Ras

## Abstract

Directional growth and tissue invasion by hyphae of the pathogenic fungus, Candida albicans, are disrupted by deletion of the small GTPase, Rsr1, which localizes Cdc42 and its kinase, Cla4, to the site of polarized growth. We investigated additional abnormalities observed in *rsr1*Δ hyphae, including vacuole development, cytoplasm inheritance, mitochondrial morphology, septin ring organization, nuclear division and migration, and branching frequency, which together demonstrate a fundamental role for Rsr1 in cellular organization. Rsr1 contains a C-terminal CCAAX box, which putatively undergoes both reversible palmitoylation and farnesylation for entry into the secretory pathway. We expressed variants of Rsr1 with mutated C244 or C245, or which lacked GTPase activity (Rsr1^K16N^ and Rsr1^G12V^), in the *rsr1*Δ background and compared the resulting phenotypes with those of mutants lacking Bud5 (Rsr1 GEF), Bud2 (Rsr1 GAP), or Cla4. Bud5 was required only for cell size and bud site selection in yeast, suggesting there are alternative activators for Rsr1 in hyphae. Septin ring and vacuole dynamics were restored by expression of unpalmitoylated Rsr1^C244S^, which localized to endomembranes, but not by cytoplasmic Rsr1^C245A^ or GTP/GDP-locked Rsr1, suggesting Rsr1 functions at intracellular membranes in addition to the plasma membrane. Rsr1^K16N^ or cytoplasmic Rsr1^C245A^ restored normal nuclear division but not septin ring or vacuole dynamics. Rsr1-GDP therefore plays a specific role in suppressing START, which can be signaled from the cytosol. Via differential palmitoylation and activity states, Rsr1 operates at diverse cell sites to orchestrate proper nuclear division and inheritance during constitutive polarized growth. As *cla4*Δ phenocopied *rsr1*Δ, it is likely these functions involve Cdc42-Cla4 activity.

## Abstract (precis)

Understanding how single eukaryotic cells self-organize to replicate and migrate is relevant to health and disease. In the fungal pathogen, Candida albicans, the small GTPase, Rsr1, guides the directional growth of hyphae that invade human tissue during life-threatening infections. Rsr1 is a Ras-like GTPase and a homolog of the conserved Rap1 subfamily, which directs migration in mammalian cells. Research into how this single GTPase delivers complex intracellular patterning is challenging established views of GTPase regulation, trafficking, and interaction. Here, we show that Rsr1 directly and indirectly coordinates the spatial and temporal development of key intracellular macrostructures, including septum formation and closure, vacuole dynamics, and nuclear division and segregation, as well as whole-cell morphology by determining branching patterns. Furthermore, we categorize these functions by differential Rsr1 localization and activity state and provide evidence to support the emerging view that the cytosolic pool of Ras-like GTPases is functionally active.

## INTRODUCTION

The morphological switch from commensal yeast to tip-growing hyphal filaments is a key virulence trait of Candida albicans, an opportunistic fungal pathogen (1). Hyphae penetrate diverse sites within the body, even in-dwelling medical devices, during superficial mucosal and life-threatening bloodstream infections (2–4). Tissue invasion by C. albicans hyphae requires the small GTPase, Rsr1, which mediates directional growth responses by stably positioning the Spitzenkörper (Spk; an accumulation of exocytic vesicles) at the hyphal tip (5–8). In yeast, the new bud site is determined by Rsr1/Bud1, which positions the guanine exchange factor (GEF), Cdc24, at the plasma membrane (PM) to activate the polarity GTPase, Cdc42, and its effector kinases, Cst20 and Cla4 (9–11). During hyphal polarization, Rsr1 positioning is not confined by intrinsic cues (5, 7, 8). C. albicans hyphae grow constitutively at the tip to form a parallel tube of mononucleate cells, which requires strict spatiotemporal organization of cellular events (12, 13). As hyphae extend, cytoplasm is asymmetrically inherited by the apical cell through the fusion and subsequent expansion of vacuoles to fill the subapical space (14). In Saccharomyces cerevisiae, the early tethering and docking stages of vacuole fusion require Cdc42-Cla4 activity (15, 16). The accumulation of cytoplasm activates the cell size checkpoint and the initiation of START by G_1_ cyclins in association with Cdc28 (17–20). START signals nuclear entry into the G_1_-S-phase transition and the simultaneous, but independent, assembly by Cdc42-Cla4 of a ring of septin filaments that define the incipient growth site (21). In C. albicans hyphae, the nascent septin ring (the presumptum) is tethered at the plasma membrane just behind the extending tip. As the tip extends, the septin ring accumulates regulators and effectors that organize septal closure after passage of a daughter nucleus into the apical cell (12, 13, 22). Septal closure involves splitting of the septin ring to make way for actinomyosin ring (AMR) contraction and synthesis of the primary (chitin) and secondary (glucan) septal walls (23, 24). The temporal coupling of nuclear inheritance with septal closure ensures the irreversible segregation of a single daughter nucleus into the hyphal tip, along with sufficient cytoplasmic resources for continued growth. The vacuolated subapical cell does not re-enter the cell cycle until sufficient cytoplasm has been regenerated to initiate a new growth axis (branch). The formation of new lateral branches is therefore confined to older cell compartments that are distal to the hyphal tip (25, 26, and reviewed in references 8 and 27). The lack of lateral growth in the vicinity of the tip is termed “apical dominance” and is common in tip-growing organisms, such as plant shoots and filamentous fungi, as it facilitates maximum tip extension with minimal investment of cytoplasm (28–30).

The Rsr1 GTPase has 59% identity to human Rap1A, an evolutionarily conserved protein that organizes cell migration (31). Rsr1 activity is localized by its GEF, Bud5, and its GTPase-activating protein (GAP), Bud2 (32). Deletion of *RSR1* caused mislocalized septins and increased hyphal branching frequency, while deletion of *RSR1* or *BUD2* caused random budding patterns and the formation of large rounded yeast cells (5, 6, 32, 33). *BUD5* deletion in C. albicans has not been characterized. Rsr1 GDP-GTP cycling localizes Cdc42 activity through isoform-specific binding—Rsr1-GTP binds Cdc24 and Rsr1-GDP binds the scaffold protein, Bem1 (32, 34–36). The Cdc42 effector, Cla4, is involved in the assembly of septin rings at the hyphal tip, and in vacuolar fusion (11, 37). In S. cerevisiae, Cla4 also initiates phosphorylation and daughter cell localization of the GEF domain protein, Lte1, until mitotic exit, although its role in C. albicans has not been elucidated (38–40).

Unlike Rsr1 in S. cerevisiae, the Rsr1 C-terminus contains a CCAAX domain with a cysteine residue (C244) adjacent to the CAAX (C, cysteine; A, aliphatic amino acid; X, any amino acid) box. This CCTIM motif suggests that Rsr1 C244 undergoes reversible palmitoylation, as occurs in C. albicans Ras1, while the terminal Met residue indicates farnesylation at C245 (41, 42). To investigate the role of C-terminal modification and activity state in Rsr1 function in C. albicans, we investigated which phenotypes of the *rsr1*Δ null mutant were rescued by expression of each of 4 Rsr1 variants carrying a mutated a posttranslational modification site, with or without an N-terminal yellow fluorescent protein (YFP) tag. Rsr1^C244S^ and Rsr1^C245A^ carried a mutation in the C-terminal Cys244 putative palmitoylation site and Cys245 CAAX box farnesylation site, respectively. The phenotypes of *rsr1*Δ mutants expressing Rsr1^K16N^ or Rsr1^G12V^, which mimicked the GDP- or GTP-locked forms of Rsr1, respectively, were compared with those of strains deleted in Bud5 (Rsr1 GEF) or Bud2 (Rsr1 GAP). We identified five phenotypic groupings, each requiring differential regulation of Rsr1. All the phenotypes exhibited by *rsr1*Δ and hyperactive Rsr1 mutants were phenocopied by deletion of *cla4*. As Cla4 acts downstream of the Rsr1 effector, Cdc42, and upstream of cortical Rsr1 via Lte1 (11, 39), we conclude that Rsr1 and Cdc42-Cla4 interactions at diverse cell sites couple the spatial organization of the cell with the ordered temporal events of the cell cycle.

## RESULTS

### Rsr1 localization depends on C-terminal Cys^244^ and Cys^245^ modification but is independent of activation state or GDP-GTP cycling.

**FIG 1:**
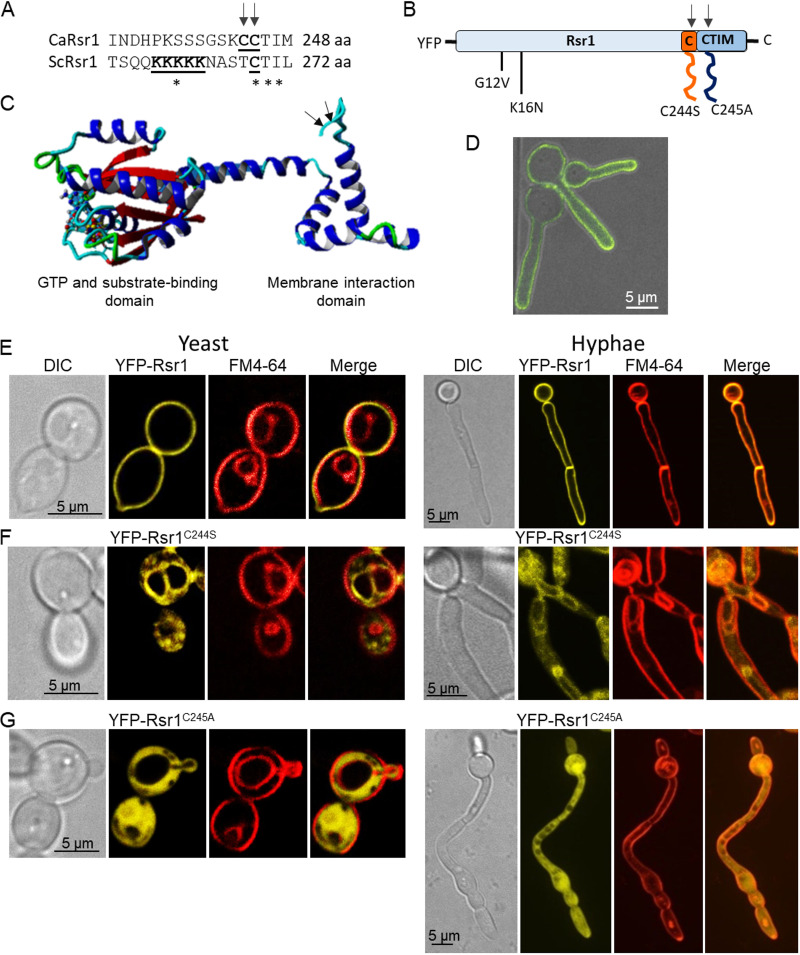
C-terminal Cys^244^ and Cys^245^ mediate differential Rsr1 membrane association. (A) Alignment of Rsr1 C termini of C. albicans and S. cerevisiae homologs. (B) Sites of Rsr1 point mutations: G12V (GTP-locked), K16N (GDP-locked), C244S (palmitoylationΔ), C245A (prenylationΔ). (C) Predicted Rsr1 3D structure showing sites of C-terminal modification (arrows), based on sequence analysis of ORF CR_02140W_A using Yasara software. (D) (see also Movie S1 in the supplemental material) Dynamic transport and PM association of YFP-Rsr1, with a bias toward the hyphal tip. (E to G) YFP-tagged Rsr1 and C-terminal mutants stained with FM4-64 in yeast and hyphae: wild-type YFP-Rsr1 (E), YFP-Rsr1^C244S^ (F), and YFP-Rsr1^C245A^ (G). Presented is medial focal plane.

Farnesylation of the C-terminal CCAAX motif is irreversible and directs nascent peptides to the endoplasmic reticulum (ER) for proteolysis of AAX and methylation (43, 44). Palmitoylation at the Golgi apparatus then promotes stable, but reversible, association with the plasma membrane via the exocytic pathway (42, 45, 46). The role of the CCAAX domain in Rsr1 localization was investigated by expressing a single copy of YFP-Rsr1, YFP-Rsr1^C244S^, or YFP-Rsr1^C245A^ in the *rsr1*Δ null background (Fig. 1A to C). The native promoter generated only a faint signal for YFP-Rsr1 and so was replaced by the *ACT1* promoter. *ACT1p*-YFP-Rsr1 rescued all *rsr1*Δ phenotypes similarly to reintegration of a single copy of *RSR1*, demonstrating that N-terminally tagged Rsr1 was functional. YFP-Rsr1 localized throughout the PMs of yeast and hyphal cells, including at septa, in a dynamic manner and was enriched toward the hyphal tip (Fig. 1D and E; see also Movie S1 in the supplemental material). Mutation of the palmitoylation site, Cys244, to serine abolished the YFP-Rsr1^C244S^ signal at the PM and localized it to endomembranes and the vacuole (Fig. 1F). Expression of Rsr1^C244S^ rescued all *rsr1*Δ phenotypes except bud site selection and agar penetration, suggesting that Rsr1^C244S^ retains function at both the PM and endomembrane, and undergoes GDP-GTP cycling (see below). Although no YFP-Rsr1^C244S^ signal was observed at the PM, farnesylation may direct Rsr1^C244S^ transiently to the PM via the normal trafficking pathway, as reported for S. cerevisiae Ras2 [ScRas2(SCaax)] (47), where it may be activated by its GEF, Bud5. Alternatively, there may be as-yet-unidentified GEFs at endomembranes that activated Rsr1^C244S^. Mutation of Rsr1^Cys245^ to alanine abolished YFP-Rsr1^C245A^ membrane association, and it became cytosolic (Fig. 1G). This is consistent with loss of the CAAX box farnesylation signal that targets proteins to the ER and secretory pathway, as seen for CaRas1 (42).

10.1128/mBio.01666-20.1 MOVIE S1 Rsr1 associates with the plasma membrane in a dynamic manner, with an intensity bias towards the hyphal tip. Time-lapse fluorescence microscopy of YFP-Rsr1 using spinning disk confocal microscope (Fig. 1D). Cells were grown in PDMS ridged chambers, and images represent a medial plane acquired at 0.161 time points per s and exported as 3 frames per s (fps). Download Movie S1, MOV file, 0.6 MB. Copyright © 2020 Bedekovic et al. 2020 Bedekovic et al. This content is distributed under the terms of the Creative Commons Attribution 4.0 International license.

**FIG 2:**
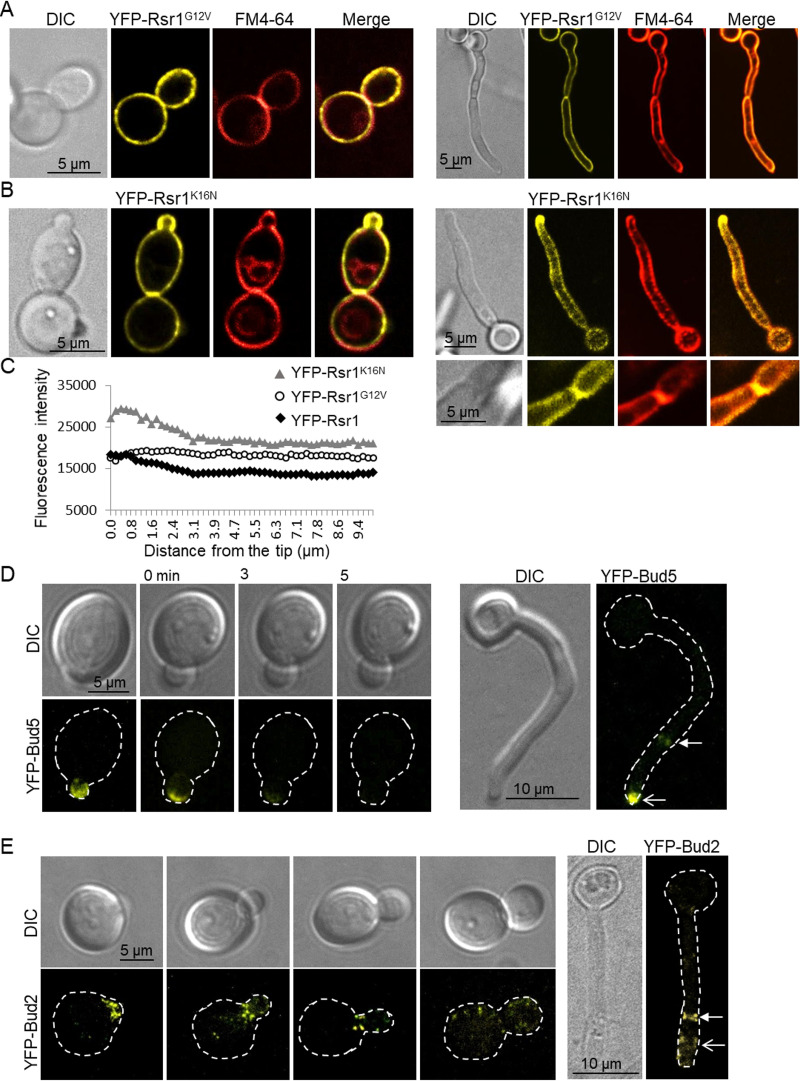
Distribution of YFP-Rsr1^K16N^ is biased toward sites of new growth. Yeast and hyphae expressing YFP-Rsr1^G12V^ (A) or YFP-Rsr1^K16N^ (B) were stained with FM4-64. (C) The fluorescence intensity across the apical 10-μm region was determined for strains YFP-RSR1, YFP-Rsr1^G12V^, and YFP-Rsr1^K16N^ using ImageJ (*n* = 40). Localization of Rsr1 GEF, Bud5 (D), and GAP, Bud2 (E), in yeast and hyphae. Closed arrows, septa; open arrows, nonseptal FP localization. Time lapse (min) indicates imaging of a single cell. Presented is medial focal plane (A, B, and C) and maximum projection of z-stacks (D and E).

To investigate whether activity state affected Rsr1 localization and function, strains expressing YFP-Rsr1^K16N^ (GDP-bound mimic) or YFP-Rsr1^G12V^ (GTP-bound mimic) were imaged (48, 49). YFP-Rsr1^G12V^ localized uniformly at the cell periphery in yeast and hyphae and was visible at septa (Fig. 2A). YFP-Rsr1^K16N^ localized throughout the PM and at septa but was biased toward the hyphal tip and appeared as small aggregates in yeast and hyphal membranes (Fig. 2B). Signal intensity analysis of the hyphal tip region showed that enrichment of YFP-Rsr1^K16N^ in the 3-μm subapical PM accounted for the overall increase in the YFP-Rsr1 signal (Fig. 2C). That Rsr1 localized to the PM in its constitutively GDP- or GTP-bound state indicated that a specific activity state or GDP-GTP cycling was not required for targeting Rsr1 to the PM. The site of Rsr1 activity in S. cerevisiae is determined by positioning of its GEF, Bud5, and GAP, Bud2 (32, 50), and so we imaged YFP-Bud5 and YFP-Bud2 in C. albicans yeast and hyphae. YFP-Bud5 was strictly confined to sites of polarized growth, with a faint signal at hyphal septa (Fig. 2D) (32). YFP-Bud2 appeared as a subapical collar around the mother bud neck in yeast, in hyphae, and at septa (Fig. 2E). The positioning of Bud5 and Bud2 therefore confines Rsr1 activity to sites of growth at the PM but raises the question as to whether all Rsr1 GDP-GTP exchanges require passage through these highly defined PM domains.

### Dysregulation of Rsr1 produced enlarged yeast cells with random budding patterns and non-invasive hyphae.

**FIG 3:**
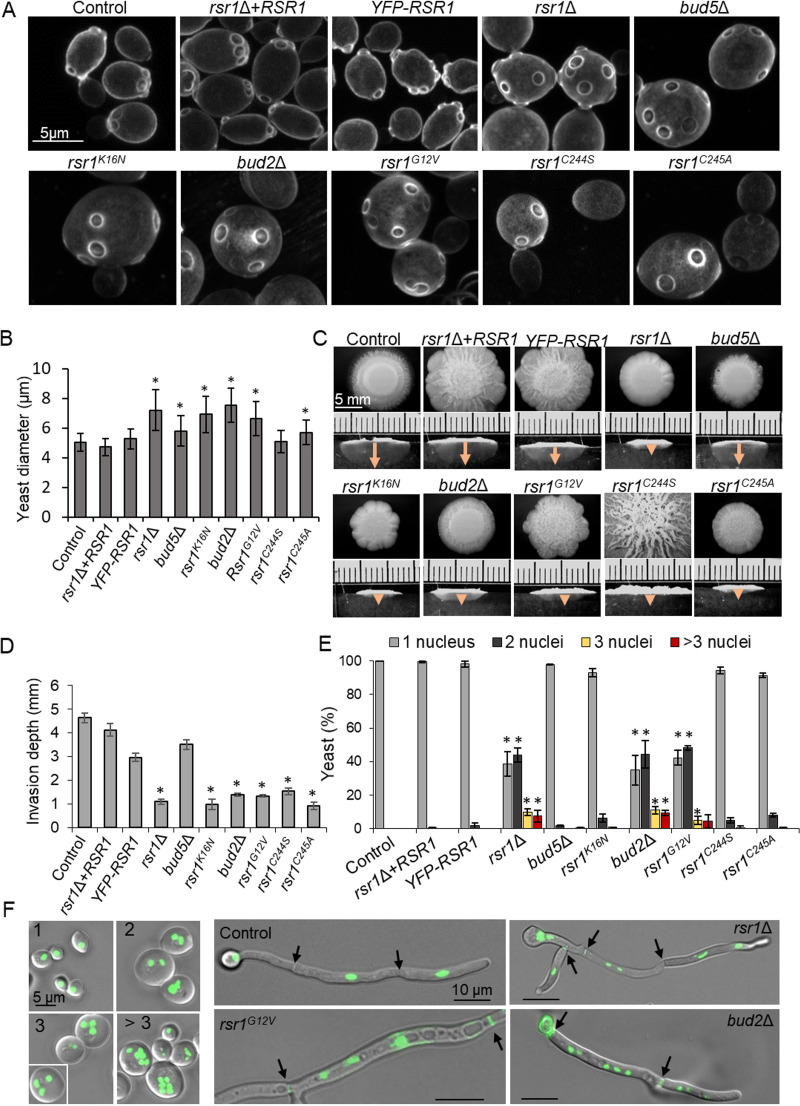
Proper regulation of Rsr1 is required for polar budding, agar invasion, and normal cell ploidy. Mutant phenotypes are also summarized in Table 1. (A) Yeast cells were grown for 16 h in YPD at 30°C and stained with CFW. (B) Yeast diameter was measured after growth for 20 h in YPD at 30°C and analyzed with a one-way analysis of variance (ANOVA) and Dunnett’s *post hoc* test. *, *P* ≤ 0.05, *n* = 100 cells per strain. (C) Agar invasion after 6 days of incubation at 37°C. Invasion depth (closed arrows) was visualized by cross-sectioning colonies. (D) Invasion depth was analyzed against control using one-way ANOVA with Kruskal-Wallis test. *, *P* ≤ 0.05, *n* = 3 (9 colonies/strain). (E and F) Yeast cells were grown for 20 h and stained with DAPI. Nuclei were quantified from maximum projections of z-stacks and analyzed using a one-way ANOVA with Bonferroni’s *post hoc* test. *, *P* ≤ 0.05, *n* = 3 (≈300 cells per strain). Septa (closed arrows) were visualized with 0.1 μg/ml CFW. Images in panel F are maximum projections of z-stacks. Error bars, standard deviations.

The *rsr1*Δ and *bud2*Δ mutants produce enlarged yeast cells with random budding patterns (5, 6). Rsr1^K16N^, Rsr1^G12V^, or Rsr1 with C-terminal modifications (Rsr1^C244S^ or Rsr1^C245A^) was expressed in the *rsr1*Δ null background, and yeast cells were compared with *rsr1*Δ, *bud2*Δ, and *bud5*Δ mutants by staining with calcofluor white (CFW). All the mutants with altered Rsr1 GDP-GTP cycling and the cytoplasmic Rsr1^C245A^ strain grew as enlarged yeast cells, and z-stack projections confirmed that bud sites were randomly positioned (Fig. 3A and B). Expression of endomembrane-localized Rsr1^C244S^ rescued the enlarged cell phenotype but was unable to restore polarized budding patterns. Normal bud site selection therefore required Rsr1 to be palmitoylated and to undergo Bud5-Bud2-mediated GTP-GDP cycling, while cell enlargement was a separable phenotype (Table 1) that was rescued by a non-palmitoylated, but otherwise functional, copy of Rsr1.

**TABLE 1:** *rsr1*Δ mutant phenotypic groupings^*a*^

Group	Phenotype	Genotype								*cla4*Δ citation
		*rsr1*Δ	*bud5*Δ	*rsr1^K16N^*	*bud2*Δ	*rsr1^G12V^*	*rsr1^C244S^*	*rsr1^C245A^*	*cla4*Δ	
1	Random bud site	1	1	1	1	1	1	1	1	This study
2	Enlarged yeast cells	1	1	1	1	1	0	1	#	11
3	Loss of agar penetration	1	0	1	1	1	1	1	1	11
4	Disorganized septins	1	0	1	1	1	0	1	1	86^*b*^, this study
4	Fragmented vacuoles	1	0	1	1	1	0	1	1	This study
4	Spk instability/loss of directionality	1	0	1	1^*c*^	1	0	1	ND	
5	Multinucleate cells	1	0	0	1	1	0	0	1	11
5	Increased secondary growth	1	0	0	1	1	0	0	1	This study
5	Hyphal bursting	1	0	0	1	1	0	P	1	87^*d*^

a Phenotypes were grouped according to their dependency on differential combinations of Rsr1 isoforms and regulators. See text for discussion. 0, WT phenotype; 1, exhibited phenotype; P, partial phenotype; ND, not determined; #, misshapen yeast cells.

b In S. cerevisiae *cla4*Δ (Sc*cla4*Δ).

c Loss of directionality, Spk not detectable at this hyphal length.

d In double Sc*cla4*Δ *cst20*Δ mutant.

*RSR1* deletion abrogates hyphal penetration of growth substrates, host cell layers, and tissue (6, 7). To test the functionality of the Rsr1 variants in substrate invasion and directional growth, hyphal penetration depth was determined after growth on hypha-inducing Spider agar, and Rsr1-mediated stability of the Spk within the hyphal tip was examined using Mlc1-YFP as a marker (8). None of the Rsr1 variants rescued the *rsr1*Δ invasion phenotype (Fig. 3C and D), indicating that hyphal invasion required stable Rsr1 palmitoylation and GDP-GTP cycling. However, the stability of Spk positioning in the apex of hyphae expressing Rsr1^C244S^ was normal (see Fig. S3). Rsr1 membrane association through farnesylation alone was sufficient to confer proper Spk positioning but not to direct substrate invasion. Bud2 was required for agar penetration and Spk positioning but Bud5 was not, even with Rsr1 GDP-GTP cycling, again suggesting an alternative activation mechanism for Rsr1. Reintegration of *RSR1* or YFP-Rsr1 in the *rsr1*Δ null restored the wild-type phenotypes in both yeast and hyphae.

### Rsr1 deletion or hyperactivation mutants generate a multinucleate phenotype that is rescued by expression of Rsr1-GDP.

Yeast cells that are large and rounded are often multinucleate (51, 52). To investigate ploidy in the mutants, the percentage of mutant yeast cell populations containing 1, 2, 3, or >3 4′,6-diamidino-2-phenylindole (DAPI)-stained nuclei was quantified. In the control, reintegrant, and YFP-Rsr1-expressing strains, yeast cells were of normal size and contained a single nucleus (Fig. 3E). In the *rsr1*Δ, *bud2*Δ, and Rsr1^G12V^ strains, ∼40% of cells contained 1 nucleus and a similar percentage contained 2 nuclei. The remaining ∼20% contained 3 to 6 distinguishable nuclei, where 6 was the limit of detection of discrete nuclei. Hyphal compartments were similarly multinucleate (Fig. 3F). Deletion or hyperactivation of Rsr1 therefore resulted in multinucleate cells. Yeast cells expressing Rsr1^K16N^, Rsr1^C244S^, or Rsr1^C245A^ were large and round with random bud sites but were mononucleate, as was the *bud5*Δ null. Cells expressing nonpalmitoylated but GDP-GTP cycling Rsr1 (Rsr1^C244S^) or GDP-bound Rsr1 (Rsr1^K16N^ or Rsr1^C245A^), irrespective of cytosolic or PM localization, were mononucleate. This indicates that Rsr1-GDP plays an isoform-specific role in the negative regulation of nuclear division.

### Multinucleate cells arise through aberrant nuclear division, migration, and segregation in the *rsr1*Δ mutant.

**FIG 4:**
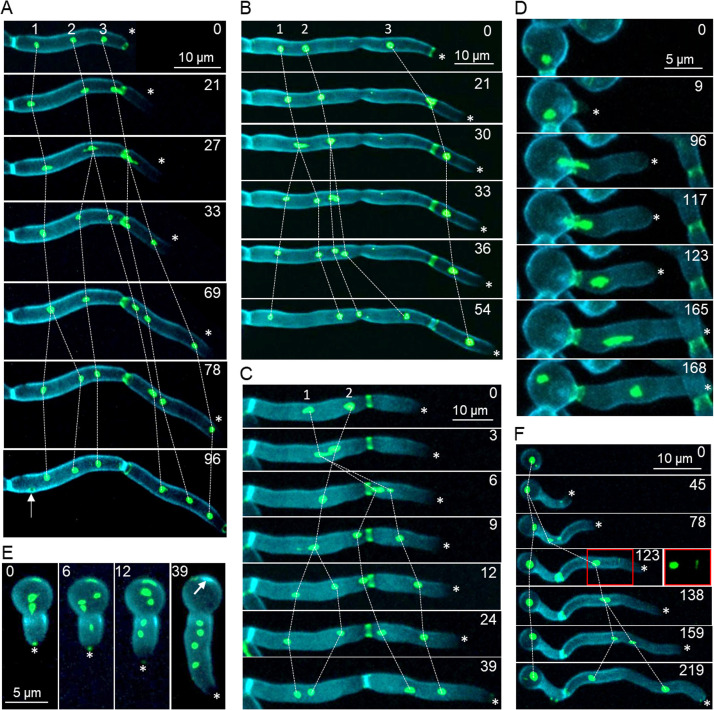
Abnormal nuclear division, migration, and inheritance in the *rsr1*Δ mutant. The temporal and spatial organization of nuclear division and migration across the presumptum were visualized using Cdc3-GFP and Nop1-YFP expressed in the *rsr1*Δ and control strains. Cell walls and primary septa were stained with CFW. Asterisks indicate new growth zones, which do not bind CFW. Minutes from the first frame (*t* = 0) are indicated. (A) Synchronous and asynchronous nuclear division. Septin ring corresponding to branch formation marked (closed arrow) at *t* = 96 min. (B) Transseptal migration of an undivided nucleus. Nonsegregation of daughter nuclei from mother cell. (C) Nuclear division in daughter cell, bidirectional daughter cell migration, and daughter cell inheritance of both nuclei. (D) Nuclear division in the daughter cell and retrograde transport of one nucleus to the mother cell. (E) Septum formation mislocalizes to mother cell cortex (arrow). (F) Incomplete septin ring formation leads to binucleate compartment (red box at *t* = 123 min).

To investigate how multinucleate cells arose in the *rsr1*Δ mutant, *RSR1* was deleted in a strain coexpressing YFP-tagged Nop1, a nucleolar protein, and Cdc3, a septin (12). This allowed visualization of nuclear division and trafficking in hyphae in relation to septum formation over several cell cycles. In *rsr1*Δ multinucleate compartments, some nuclei divided synchronously in pairs, suggesting both responded to a single START signal (Fig. 4A, nuclei 2 and 3; Movie S2; Fig. 4B, nuclei 1 and 2; Movie S3), while others divided asynchronously, suggesting START was initiated as two separate events (Fig. 4A; Movie S2; Fig. 4C, nuclei 1 and 2; Movie S4). Five abnormal nuclear migration patterns were observed in the mutants. First, a nucleus migrated from the mother compartment across the presumptum into the daughter cell without having first undergone division (Fig. 4B; Movie S3, Fig. 4D; Movie S5). Second, both daughters of a newly divided nucleus migrated through the presumptum and remained in the daughter compartment (nucleus 3 in Fig. 4A; Movie S2). Third, a daughter nucleus migrated across the septum into the mother cell and back into the daughter cell (Fig. 4C; Movie S4). Fourth, a nucleus migrated across the presumptum before dividing in the daughter cell, whereupon one daughter nucleus migrated back into the mother cell (Fig. 4D; Movie S5). Fifth, nuclei underwent division but neither daughter migrated to the apical compartment (Fig. 4A to C; Movies S2, S3, and S4). Multinucleate cells also arose due to the failure of septa to form an adequate cell boundary, either through mislocalization of septins at the mother cell cortex (Fig. 4E) or through incomplete septum formation (Fig. 4F).

10.1128/mBio.01666-20.2 MOVIE S2 Synchronous and sequential nuclear division in the *rsr1*Δ mutant. Time-lapse fluorescence microscopy of two nuclei (Nop1-GFP) that undergo synchronous nuclear division. Three daughter nuclei migrate through the presumptum (Cdc3-GFP) into the daughter compartment. Asynchronous division occurs in the mother compartment, but neither daughter migrates (Fig. 4A). Cells were stained with 2 μg/ml CFW. Images are maximum projections of z-stacks acquired every 3 min and exported as 3 fps. Download Movie S2, MOV file, 0.3 MB. Copyright © 2020 Bedekovic et al. 2020 Bedekovic et al. This content is distributed under the terms of the Creative Commons Attribution 4.0 International license.

10.1128/mBio.01666-20.3 MOVIE S3 Nuclear inheritance without division in the *rsr1*Δ mutant. Time-lapse fluorescence microscopy of nuclear division in trinucleate hyphal compartment and migration of nondividing nucleus to the apical daughter compartment. The other 2 nuclei divide synchronously, but no nuclei migrate into the daughter cell (Fig. 4B). Cells were stained with 2 μg/ml CFW. Images are maximum projections of z-stacks acquired every 3 min and exported as 3 fps. Download Movie S3, MOV file, 0.1 MB. Copyright © 2020 Bedekovic et al. 2020 Bedekovic et al. This content is distributed under the terms of the Creative Commons Attribution 4.0 International license.

10.1128/mBio.01666-20.4 MOVIE S4 Sequential nuclear division and aberrant migration in the *rsr1*Δ mutant. Time-lapse fluorescence microscopy of two daughter nuclei from the first division entering the daughter cell. One temporarily migrates into the mother cell before re-entering the daughter cell, followed by septal closure. The daughter nuclei from the second nuclear division are retained in the mother compartment (Fig. 4C). Cells were stained with 2 μg/ml CFW. Images are maximum projections of z-stacks acquired every 3 min and exported as 3 fps. Download Movie S4, MOV file, 0.1 MB. Copyright © 2020 Bedekovic et al. 2020 Bedekovic et al. This content is distributed under the terms of the Creative Commons Attribution 4.0 International license.

10.1128/mBio.01666-20.5 MOVIE S5 Nuclear division in the daughter cell in the *rsr1*Δ mutant. Time-lapse fluorescence microscopy of the nucleus (Nop1-GFP) migrating through the septin ring (Cdc3-GFP) prior to nuclear division followed by the retrograde migration of a daughter nucleus into the mother cell (Fig. 4D). Cells were stained with 2 μg/ml CFW. Images are maximum projection of z-stacks acquired every 3 min and exported as 3 fps. Download Movie S5, MOV file, 0.4 MB. Copyright © 2020 Bedekovic et al. 2020 Bedekovic et al. This content is distributed under the terms of the Creative Commons Attribution 4.0 International license.

Others have reported multinuclear phenotypes that we did not observe; thus, we can exclude the mechanisms involved. First, nuclei did not divide in cell compartments that were bounded by closed septa unless accompanied by the emergence of a new branch, and so the nuclear division and cell polarization signals downstream of START both initiated as normal. Second, anucleate cells did not arise, which excluded a failure to detect correct spindle alignment along the polarit*y* axis, as seen in the *cdc10*Δ mutant (53, 54). Third, although single daughter nuclei underwent retrograde migration into the mother cell, we did not see both daughter nuclei translocate in this way. This suggests that elongation of the mitotic spindle was normal in the *rsr1*Δ mutants, as this drives retrograde movement of the daughter nucleus that is retained by the mother cell (12). Finally, septal closure did not occur without the transfer of at least one nucleus into the daughter cell, suggesting that the signal to undergo AMR contraction remained coupled to nuclear migration.

Multinucleate cells therefore arose through at least three mechanisms: nuclear division with failed/mislocalized septum formation, multiple nuclear division events within single hyphal compartments (prior to septal closure) without daughter nucleus segregation, and aberrant migration through the presumptum of either undivided nuclei or both daughter nuclei, followed by septal closure.

### Deletion of Rsr1 caused defective septin ring localization, structure, and separation.

**FIG 5:**
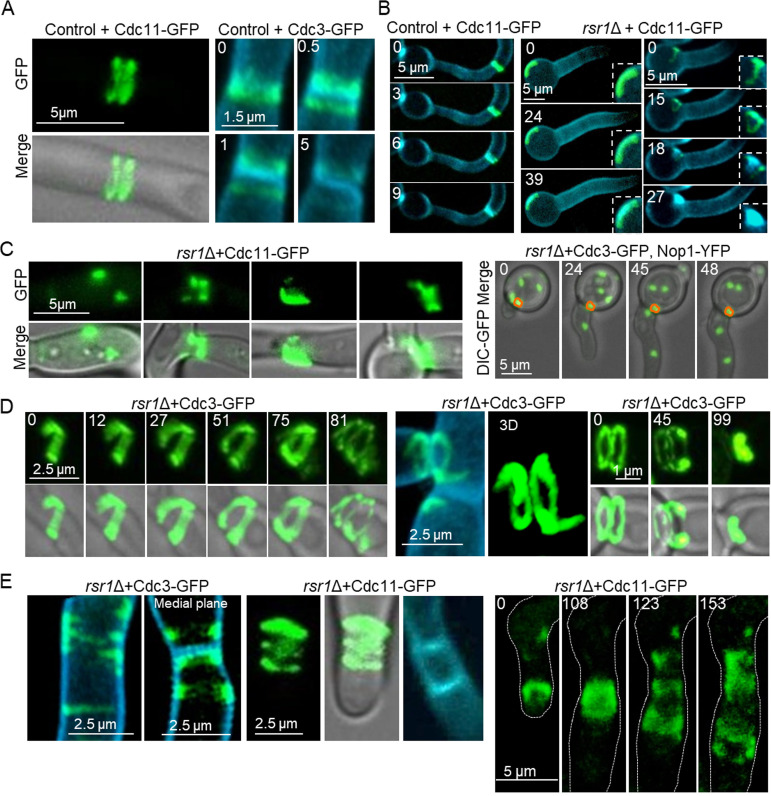
Abnormal septin ring assembly and localization. Cdc11-GFP and Cdc3-GFP were imaged during septin ring assembly, septin ring separation, and primary septum formation in hyphae of the control strain and the *rsr1*Δ mutant. Time lapsed (min) from the first frame is indicated. (A) Septin ring separation imaged using Cdc11-GFP or Cdc3-GFP in wild-type hyphae. (B) Cdc11-GFP and chitin localized at hyphal septa in wild-type cells but within the basal mother cell in the *rsr1*Δ mutant. (C) Ectopic localization of Cdc11- and Cdc3-GFP within hyphal compartments. Outlined (right) is aberrant septin structure formation in the *rsr1*Δ+Cdc3-GFP, Nop1-YFP strain. (D) Ring formation and separation in the *rsr1*Δ mutant expressing Cdc3-GFP. (E) Formation of multiple septin rings in the *rsr1*Δ mutant gave rise to double primary septa. Septins form diffuse triple rings in the *rsr1*Δ mutant. Images are a maximum projection of z-stacks.

Septin ring assembly requires the iterative activity of Cdc42-Cla4 (21, 37). As septin ring behavior was compromised in the *rsr1*Δ mutant, septin ring morphology, localization, and behavior were examined by imaging Cdc3-GFP or Cdc11-GFP in the *rsr1*Δ mutant and wild-type cells during hyphal growth (Cdc11-GFP and Cdc3 are functional in C. albicans [12, 55]). In wild-type cells, a single septin ring formed within hyphae and, after nuclear migration, split in two to provide space for AMR contraction and synthesis of the primary septum (visualized using CFW) (Fig. 5A). In *rsr1*Δ hyphae, septins occasionally formed an ectopic primary septum at the mother cell cortex. This suggested that septin ring formation had been initiated by START signaling, but lack of the Rsr1 positioning signal allowed septins to assemble at random cell sites instead of within the hyphal tip (Fig. 4E and 5B). Septin rings also coalesced into an immobile cortical patch at the hyphal periphery, suggesting the hetero-octamers were unable to maintain higher order ring structure (Fig. 4F and 5C). In the *rsr1*Δ-Cdc3-GFP strain, septins appeared as short spirals or incomplete rings, which formed 3 diffuse and incomplete rings on separation and generated 2 parallel primary septa (Fig. 5D and E). This is consistent with the observation that two START signals were initiated consecutively in one compartment (Fig. 4A) to produce asynchronous division of two nuclei and two septa.

**FIG 6:**
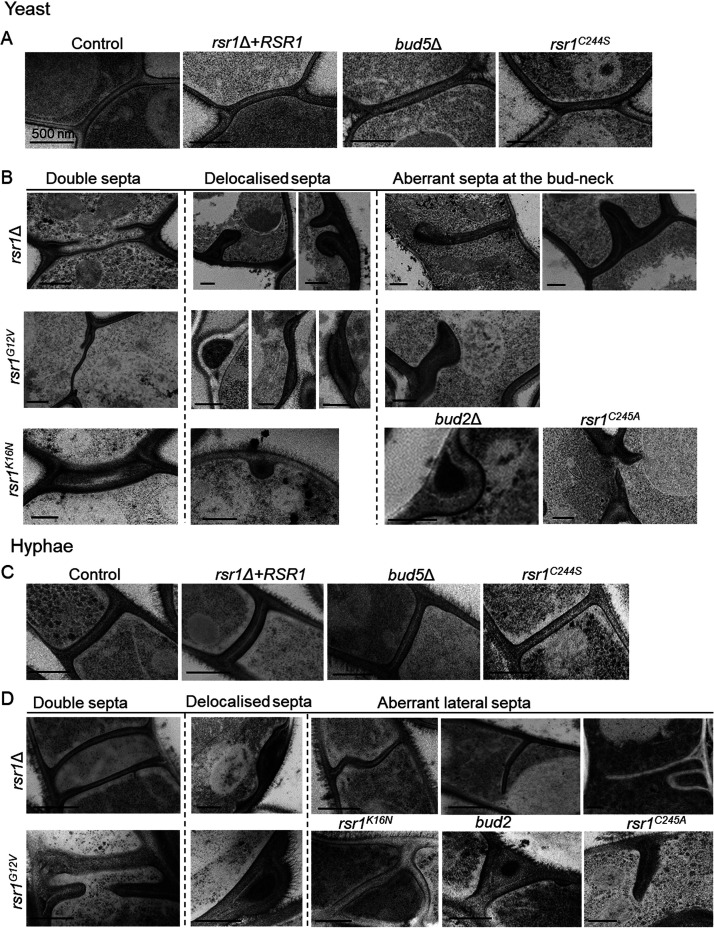
Deletion or dysregulation of Rsr1 causes aberrant septum formation. Transmission electron microscopy (TEM) of septal ultrastructure in yeast (A and B) and hyphae (C and D). (A) Normal septum morphology in the control, *RSR1* reintegrant, *bud5*Δ, and *rsr1^C244S^* strains. (B) Double septa, delocalized septa, and aberrant septa at the mother-daughter bud neck in all other mutants. (C) Normal septal structure in hyphae of the control, *RSR1* reintegrant, *bud5*Δ, and *rsr1^C244S^* strains. (D) Double septa and aberrant lateral septa in hyphae in all other mutants. Delocalized septa in mother yeast cells of the *rsr1*Δ and *rsr1^G12V^* mutants during growth as hyphae. Scale bars, 500 nm.

The ultrastructure of septa was examined in all the Rsr1 mutant strains by using transmission electron microscopy. In yeast and hyphae of the control strains and the *bud5*Δ mutant, the localization and morphology of septa were normal, with a thin, white, chitinous primary septum and the darker β-glucan layer of the secondary septum on either side (Fig. 6A and C). Septa of the *bud2*Δ and *rsr1*Δ mutants showed severe structural deformities, including incomplete or distorted septa and double septa (Fig. 6B and D) and septa that were mislocalized, appearing as in-growths of cell wall material or as “blisters” at the cell cortex, consistent with the patterns formed by FP-tagged septins (see Fig. S1). Septum morphology was rescued by expression of endomembrane-localized Rsr1^C244S^ but not by expression of Rsr1^G12V^, Rsr1^K16N^, or cytoplasmic Rsr1^C245A^. Together, these results indicate that normal septum formation and localization required Rsr1 to undergo GTP-GDP cycling, although the Rsr1 GEF, Bud5, was not required. As expression of Rsr1^C244S^ rescued the septin ring phenotype, unpalmitoylated Rsr1 appears to retain membrane-associated function and undergo GDP-GTP cycling, perhaps by transient association with the plasma membrane or by activation at internal membranes by an unknown GEF. Taken together, these results show that Rsr1 plays a role in determining the site and higher order structure of Cdc42-Cla4-mediated septin ring assembly.

10.1128/mBio.01666-20.6 FIG S1 Composite of septal phenotypes in DAY185 and *rsr1*Δ strains. TEM, fluorescence, or DIC microscopy images of normal septum morphology of the DAY185 control strain visualized by Cdc11-GFP and CFW (A) compared to images from the *rsr1*Δ strain (B). Presented is the medial focal plane. Download FIG S1, TIF file, 1.2 MB. Copyright © 2020 Bedekovic et al. 2020 Bedekovic et al. This content is distributed under the terms of the Creative Commons Attribution 4.0 International license.

To confirm the role of Rsr1 in septin ring organization, glutathione transferase (GST)-6×His-Rsr1^1–243^, a truncated version of Rsr1 lacking the C-terminal membrane localization domain, was heterologously expressed in Escherichia coli and used in pulldown assays with C. albicans lysates from yeast and hyphal cells. Cdc42 GTPase and its GEF, Cdc24, were identified, as expected. The 5 core septins—Cdc3, Cdc11, Cdc12, Cdc10, and Sep7/Shs1—were detected at levels above that for the GST control in hyphae, although Cdc10 was not seen at a level greater than that of the negative control in yeast (see Fig. S2A), confirming the association of Rsr1 with these septins through direct or indirect binding. Bud5 and Bud2 were not detectable in pulldown assays, but association of these regulators with Rsr1 was confirmed by far-Western blot (Fig. S2B).

10.1128/mBio.01666-20.7 FIG S2 Rsr1 interacting partners. (A) Interacting partners were pulled down with heterologously expressed GST-Rsr1^1–243^, lacking the C-terminal CAAX box, in yeast and hyphae and identified by mass spec. Data are presented as a ratio of signal against GST tag alone (background), *n* = 3. (B) Interaction of GST-Rsr1 with YFP-Bud5 and YFP-Bud2 was examined with a far-Western blot. After the treatment of the PVDF membrane with far-Western blot buffers, proteins were detected with anti-GFP antibody or incubated with purified GST-Rsr1 and detected with anti-GST antibody. Binding to the YFP tag alone was determined using a GFP control. Download FIG S2, TIF file, 0.8 MB. Copyright © 2020 Bedekovic et al. 2020 Bedekovic et al. This content is distributed under the terms of the Creative Commons Attribution 4.0 International license.

10.1128/mBio.01666-20.8 FIG S3 Spk stability in the hyphal tip. Aberrant sequential positioning of Mlc1-YFP during hyphal growth in PDMS chambers was visualized (A) and quantified (B). Aberrant Mlc1-YFP localization was defined as (i) nonlinear sequential positioning of Mlc1-YFP and (ii) loss of tip contact with the ridge, 15 ≤ *n* ≤ 26. Presented is the medial focal plane. Download FIG S3, TIF file, 0.8 MB. Copyright © 2020 Bedekovic et al. 2020 Bedekovic et al. This content is distributed under the terms of the Creative Commons Attribution 4.0 International license.

### Diffuse Dbf2 association with septin rings delayed AMR contraction in the *rsr1*Δ mutant.

**FIG 7:**
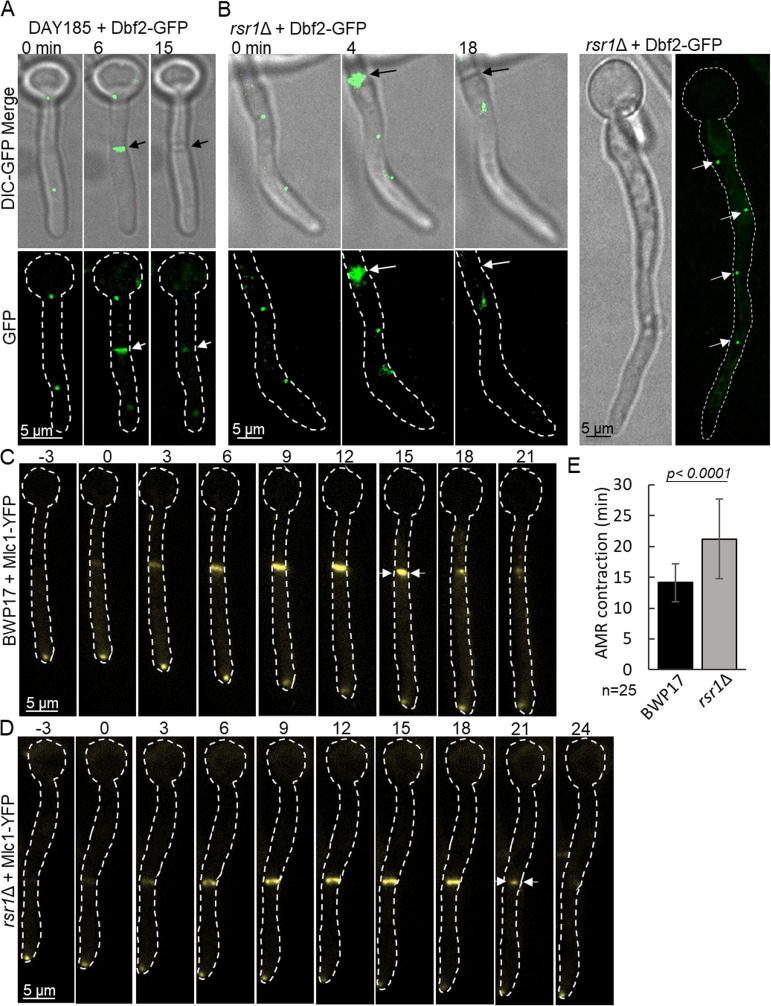
Dbf2-GFP is mislocalized and AMR contraction is delayed in the *rsr1*Δ mutant. (A) Time course of Dbf2-GFP translocation from SPBs to the septum in the DAY185 control strain. (B) Time course in the *rsr1*Δ mutant of Dbf2-GFP translocation from multiple SPBs to a diffuse cloud at the septum. Time course of AMR contraction in wild-type (C) and *rsr1*Δ (D) cells expressing Mlc1-YFP. Medial focal plane images were acquired at 3-min intervals. Time zero, first appearance of Mlc1-YFP at the septum. Arrows indicate onset of AMR contraction. (E) Difference in AMR contraction time course, *n* = 25. Unpaired *t* test with Welch’s correction (*P* < 0.0001, *n* = 25, error bars are SDs). Panels A and B show maximum projections of z-stacks.

In wild-type cells, nuclear migration through the presumptum is immediately followed by AMR contraction and septum formation. The observation that nuclei cross the septal plane bidirectionally in the *rsr1*Δ mutant suggested that the signal coupling septal closure with nuclear segregation was compromised or delayed in this mutant. The septin collar is a scaffold for 83 septin-associated proteins that integrate multiple signals to coordinate downstream events in cytokinesis (56). A key mediator of AMR contraction and primary septum formation is the essential Lats/nuclear Dbf2-related (NDR) kinase, Dbf2. Dbf2 translocates from the spindle pole bodies (SPBs) in late mitosis to associate with the separated septin rings after anaphase, where it phosphorylates Hof1, an F-BAR protein, and Chs2 to effect compartment closure (57–60). We hypothesized that Dbf2 localization might be compromised by the disorganization of septins in the *rsr1*Δ mutant. We therefore expressed C-terminally tagged Dbf2-GFP in wild-type cells and the *rsr1*Δ mutant (Dbf2-GFP functionality was demonstrated in reference 57). In wild-type cells, Dbf2-GFP localized to the SPBs of dividing nuclei, appearing as 2 bright dots aligned along the hyphal axis (Fig. 7A). Over a period of ∼12 min, the spots faded and Dbf2-GFP appeared transiently at the septum. In the *rsr1*Δ mutant, Dbf2-GFP appeared as several pairs of bright dots aligned along the hyphal axis, consistent with its association with multiple SPBs in this multinucleate mutant, but formed a diffuse cloud around the presumptum (Fig. 7B). Although disorganized, the septins could therefore recruit Dbf2 to some extent, suggesting that effector association with septin rings was not completely abrogated. Nevertheless, when we visualized Mlc1-GFP, myosin light chain 1, across the septum during AMR contraction, it was visible for 14 ± 3 min in the control strain, but for 21 ± 6 min in *rsr1*Δ hyphae, before contracting to a central spot at septal closure (Fig. 7E). AMR contraction was therefore delayed at disorganized septa, which remained open for ∼50% longer than in wild-type cells (Fig. 7C to E) and suggests that the coordination of the activity of effectors by malformed septin rings was compromised.

### Fragmented vacuoles failed to fuse and enlarge in *rsr1*Δ hyphae, leading to retention of cytoplasm and mitochondria in subapical cells.

**FIG 8:**
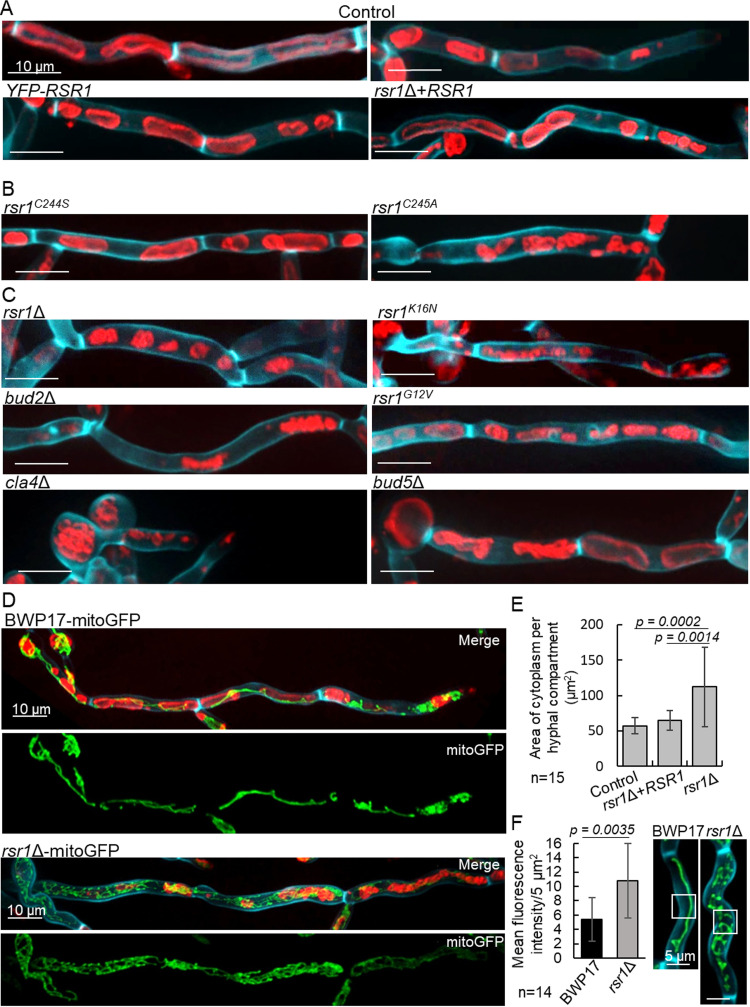
Rsr1 is required for normal vacuole and mitochondrial morphology. Membranes were stained with FM4-64, and cell wall and septal chitin were stained with CFW. (A, B, and C) Vacuole morphology in subapical hyphal compartments without branches. (D) Mitochondrial morphology visualized using mitoGFP in wild-type cells and in the *rsr1*Δ mutant, stained as described above. (E) Cytosolic area per compartment was determined by subtracting vacuole area from total compartment area by manual tracing of 2D images in z-stack maximum projections. Data were analyzed using a one-way ANOVA with Dunnett’s *post hoc* test. (F) Fluorescence intensities of mitoGFP were analyzed in areas of 5 μm^2^ central to hyphal compartments using an unpaired *t* test with Welch’s correction. Images are maximum projections of z-stacks. *, *P* ≤ 0.05; error bars, SDs; *n* values are indicated.

In S. cerevisiae, Cdc42-Cla4 activity is required for the tethering and docking stages of vacuolar fusion (16, 61, 62). In addition to Cdc42, our pulldown assays detected 5 proteins involved in vacuole priming, docking, and fusion—Sec18, Ykt6, Vac8, Vtc3 and Vtc4 (Fig. S2A)—suggesting that Rsr1 also directs Cdc42 activity during vacuole fusion in C. albicans. To investigate this further, we imaged mutant hyphae stained with the fluorescent lipid membrane dye, FM4-64. In wild-type hyphae, YFP-Rsr1 and the Rsr1 reintegrant, vacuoles in subapical G_1_ cells were enlarged and elongated, occupying ∼56% and 46% of the compartmental space, respectively (Fig. 8A), while in apical cells, newly inherited vacuoles were fragmented and had not yet undergone fusion or enlargement. In the *rsr1*Δ null, vacuoles were fragmented in apical and subapical cells alike, regardless of cell cycle stage, suggesting that vacuolar inheritance was normal but none had undergone fusion (Fig. 8C). Comparison of the contents of subapical compartments showed that vacuoles occupied only ∼36% of the cell compartment, leading to a 45% increase in cytosolic volume in *rsr1*Δ cells compared to that in the wild type (*P* ≤ 0.0002) (Fig. 8E). The *rsr1*Δ phenotype was shared by the *bud2*Δ mutant and was not rescued by expression of Rsr1^K16N^, Rsr1^G12V^, or cytoplasmic Rsr1^C245A^ (Fig. 8B and C). However, expression of Rsr1^C244S^ restored normal vacuole morphology, providing supporting evidence that this Rsr1 isoform can undergo GDP-GTP cycling and was functional at endomembranes. As Cdc42-Cla4 activity is required for vacuole fusion in S. cerevisiae, we imaged vacuoles in the *cla4*Δ mutant and found the same fragmented phenotype (Fig. 8C). Together, these results suggest that normal vacuole morphology involves Rsr1 regulation of Cdc42-Cla4 and requires Rsr1 to associate with membranes and undergo GTP-GDP cycling.

The retention of cytoplasm in subapical cells suggested they may also retain metabolic activity. In the growing apical cells of wild-type hyphae, mitochondrial structure is branched and complex, but in older G_1_-phase compartments, mitochondria are small and peripherally localized until sufficient cytoplasm is synthesized to initiate START (63). The complexity of mitochondrial morphology may therefore be an indicator of the metabolic state of the cell. We investigated mitochondrial morphology by expressing and imaging mitoGFP (mtGFP) in the *rsr1*Δ mutant (64). In G_1_ compartments of wild-type hyphae, mitochondria took the form of a thin filament at the periphery of each compartment between the cell cortex and the large vacuoles, visualized by FM4-64 staining, while mitochondria in growing apical cells were reticulate and densely packed around the fragments of newly inherited vacuole (Fig. 8D). In contrast, mitochondria in all compartments of the *rsr1*Δ mutant had the dense reticulate appearance of those in the apical cell. The quantified signal intensity in *rsr1*Δ subapical compartments was double that of wild-type cells, indicating that mitochondria occupied significantly more of the cytoplasmic volume (Fig. 8D and F). Together, these results suggest that mitochondria in the subapical cells of the *rsr1*Δ mutant were metabolically active and therefore unlikely to be in early G_1_ phase.

### Multiple secondary growth axes indicate loss of apical dominance in the *rsr1*Δ and hyperactive Rsr1 mutants, which is rescued by expression of Rsr1-GDP.

**FIG 9:**
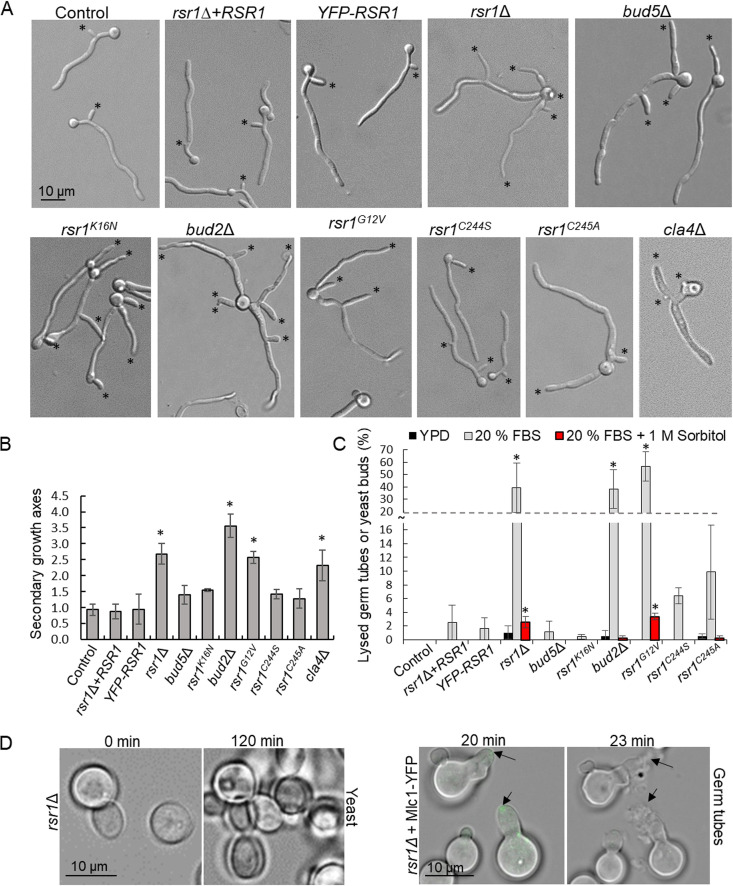
Rsr1 dysregulation caused hyperbranching and bursting. (A and B) Hyphae were induced in 20% FCS for 5 h and the number of secondary growth axes (asterisks) was determined. For *rsr1*Δ, *rsr1^K16N^*, *bud2*Δ, *rsr1^G12V^*, and *cla4*Δ mutants, optical sections were acquired for accuracy. (C and D) Cell bursting (%) was quantified in budding yeast (YPD, 30°C) (D, left) and germinating hyphae (20% FCS with/without 1 M sorbitol, 37°C, right). (Right) The Spk was imaged using Mlc1-YFP as a marker (arrows). (B and C) Data were analyzed using a one-way ANOVA with Dunnett’s *post hoc* test. *, *P* ≤ 0.05; error bars, SDs; *n* = 3 (66 cells/strain for panel B, ≈400 cells/strain for panel C).

C. albicans hyphae exhibit apical dominance, where new lateral growth (branching) is confined to older cells after sufficient uptake of nutrients for re-entry into the cell cycle, i.e., in cells that are furthest from the growing tip (25). To further probe the link between subapical metabolic activity and the prevalence of multinucleate cells, we quantified the emergence of new secondary growth axes as an indicator of entry into START. In hyphae of the 3 control strains, and in *bud5*Δ after 5 h of growth few secondary growth axes had formed (range, 0 to 2), and they emerged from the oldest cell (Fig. 9A and B). In *bud2*Δ and *rsr1*Δ mutants, multiple polarity axes (range, 1 to 9) were formed within the same time period, primarily from the oldest cell but some from younger compartments. The same values were determined in the *cla4*Δ mutant. Thus, multiple distal compartments in these mutants initiated START compared to that in the control strain, a hallmark of loss of apical dominance, and confirmed that these cells retained metabolic activity. Interestingly, the total length of hyphae produced per mother yeast cell by these mutants was greater than the wild-type strain (see Fig. S4). However, this rate of growth was unsustainable at the population level, as 40% to 55% of hyphal germination events resulted in cell lysis (*P* ≤ 0.0001). Hence, there was no net increase in overall growth, as increased hyphal extension in one subpopulation of cells was countered by a high rate of bursting and loss of viability in another (Fig. 9C and D andS4). The lysis phenotype was hypha specific, as only 4% of yeast cells burst during bud emergence. Hyphal cell bursting was rescued by the presence of 200 mM sorbitol in the medium (Fig. 9C). This implies that cell wall integrity was compromised during hyphal morphogenesis, in which initiation of true hyphal growth was confirmed by visualization of Mlc1-GFP, a reporter for Spk formation and positioning (Fig. 9D and S3) (26). As Rsr1 focuses the delivery of exocytic vesicles containing cell wall biosynthesis machinery and structural components to the hyphal tip, which are strongly associated with septin organization, the diffuse dispersal of these elements may compromise cell wall assembly (32, 65, 66).

10.1128/mBio.01666-20.9 FIG S4 Morphological changes at the whole-cell level. Rsr1 dysregulation resulted in longer hyphae. (A) Total hyphal length, determined as a sum of primary hyphal filament and lateral branches, was measured after growth in 20% FCS for 5 h. For *rsr1*Δ, *rsr1^K16N^*, *bud2*Δ, *rsr1^G12V^*, and *cla4*Δ mutants, optical sections were acquired for accuracy. Data were analyzed using a one-way ANOVA with Dunnett’s *post hoc* test. *, *P* ≤ 0.001. (B) Net hyphal growth at the population level in WT, Rsr1 reintegrant, and hyperbranching mutants after cell lysis. (C) Aberrant septum localization and cell morphology of *cla4*Δ yeast. Yeast cells were grown for 16 h in YPD at 30°C and stained with CFW. Aberrant septa are marked with closed arrows. Download FIG S4, TIF file, 0.6 MB. Copyright © 2020 Bedekovic et al. 2020 Bedekovic et al. This content is distributed under the terms of the Creative Commons Attribution 4.0 International license.

Loss of apical dominance was not rescued by expression of Rsr1^G12V^, consistent with the findings for the *bud2*Δ mutant, but was instead rescued by expression of Rsr1^K16N^, Rsr1^C244S^, or Rsr1^C245A^. Expression of Rsr1^C244S^ had already been shown to restore normal vacuole morphology, cytoplasm distribution, and septin ring organization, which are prerequisites for supporting apical dominance. However, expression of GDP-locked Rsr1^K16N^ or cytoplasmic Rsr1^C245A^ was specifically able to restore apical dominance, even though the cells retained fragmented vacuoles and increased cytoplasm. This strongly suggests that the Rsr1-GDP isoform acts as a negative regulator of START.

## DISCUSSION

**FIG 10:**
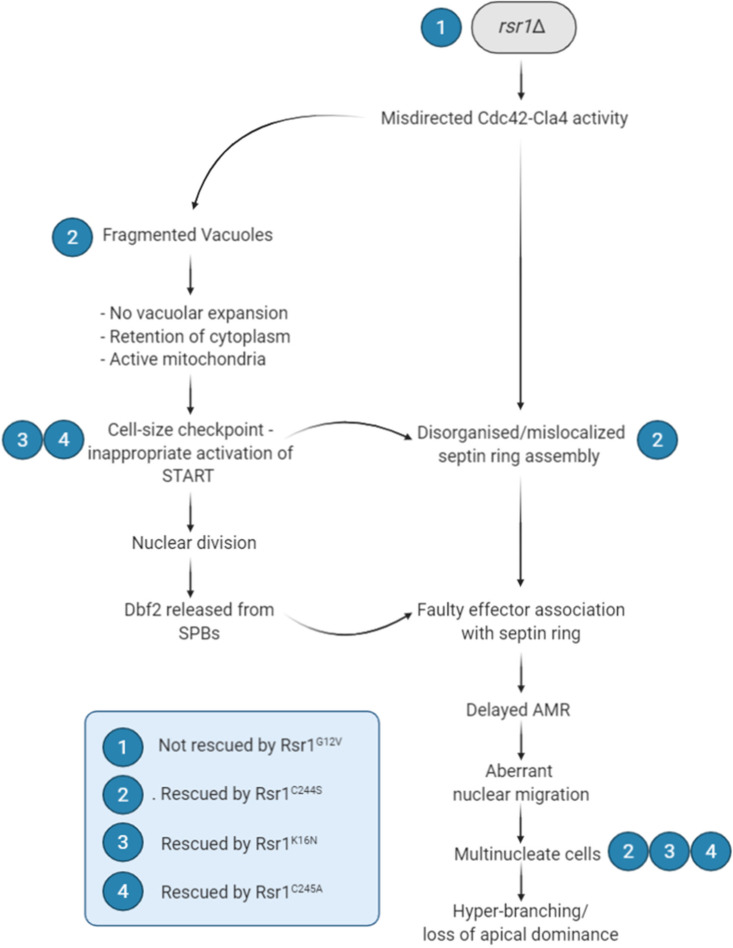
Rsr1 plays a pivotal role in the maintenance of mononucleate cells and apical dominance by C. albicans hyphae. Loss of Rsr1 function at vacuoles abolished Cdc42-mediated vacuolar fusion and expansion, a process that normally drives the asymmetric inheritance of cytoplasm by the apical cell. Retention of cytoplasm correlated with the presence of complex, likely active, mitochondria and the early onset of START in subapical cells. Disorganized and mislocalized septin ring assembly allowed the formation of a cytoplasmic continuum with aberrant nuclear migration and segregation patterns. Combined, these abnormalities resulted in multinucleate cells and hyperbranching, a defining characteristic of loss of apical dominance in tip-growing cells. Expression of endomembrane-localized Rsr1^C244S^ rescued these phenotypes. Rescue of inappropriate activation of START by Rsr1 GDP-bound isoforms, Rsr1^K16N^ or Rsr1^C245A^, was sufficient to restore apical dominance, despite the presence abnormal vacuolar and septin ring phenotypes.

This study reveals Rsr1 involvement in diverse spatial and temporal functions that are crucial for growth and development in the polymorphic fungus, C. albicans (Fig. 10), all of which likely operate through the Cdc42-Cla4 signaling module, as the observed *rsr1*Δ phenotypes were phenocopied by deletion of Cla4 and/or are linked to known Cla4 activity (Table 1). Furthermore, the multipurpose functions of Rsr1 signaling are separable by dependency on the posttranslationally modified state. Unexpectedly, Rsr1 exerted function-specific activity when localized to the cytoplasm, raising the possibility that this is a legitimate site of Rsr1 signaling, as is seen for Ras1 in C. albicans and Drosophila melanogaster (42, 67). We classified the phenotypes caused by deletion or mutation of the components of the Bud5-Rsr1-Bud2 signaling module into 5 groups, depending on their rescue by specific isoforms, regulators, or combinations thereof (Table 1).

### The canonical Bud5-Rsr1-Bud2 signaling module is required only for proper bud site selection and yeast cell morphology.

Group 1 consisted of a single *rsr1*Δ phenotype—random bud site selection in yeast—which could not be rescued by expression of any of the Rsr1 mutant constructs. Additionally, all components of the well-characterized Bud5(GEF)-Rsr1-Bud2(GAP) signaling module (50) and Cla4 were required for the wild-type phenotype. Surprisingly, no phenotypes in this class were seen in hyphae.

In group 2, enlarged yeast cell morphology was the only other phenotype we identified for the *bud5*Δ null (see below “The GEF Bud5 is largely dispensable for Rsr1 function”). Although the group 2 phenotype was Bud5 dependent, it was rescued in the *rsr1*Δ mutant by expression of unpalmitoylated Rsr1^C244S^. All other components of the Rsr1 signaling module were required for the wild-type morphology, implying that, although visualized only at endomembranes, Rsr1^C244S^ must be able to under GDP-GTP cycling. As Bud5-GFP resided at the cell periphery and Bud5 was required for normal yeast cell morphology, it is plausible that farnesylated Rsr1^C244S^ was trafficked to the PM where it was activated. The similarly modified Ras1^C288/7S^ isoform was reported at the cell periphery in C. albicans, indicating that this trafficking route is possible in farnesylated but unpalmitoylated GTPases (42). The current model for N-Ras cycling is that depalmitoylated N-Ras is trafficked to endomembranes from the PM by cytoplasmic chaperones and is not delivered directly from the Golgi apparatus (68). Without further investigation of the Rsr1^C244S^ trafficking pathway to endomembranes, it is not clear whether Rsr1^C244S^ rescued normal cell morphology through transient association with the PM or whether rescue was effected from endomembranes. *cla4*Δ yeast cells did not exhibit an increase in cell diameter but were misshapen, with elongated bud necks and mislocalized, sometimes incomplete, septa (see Fig. S4C in the supplemental material), consistent with the involvement of Cla4 in septin ring assembly and positioning.

### The GEF Bud5 is largely dispensable for Rsr1 function.

Group 3 again consisted of a single phenotype, loss of agar penetration by hyphae, where normal invasive growth required wild-type (WT) Rsr1, Bud2, GDP-GTP cycling, and the Cla4 kinase (11). It was independent of Bud5 and was not rescued by any Rsr1 isoform, including Rsr1^C244S^, the isoform that restored normal yeast cell morphology and 6 other phenotypes (discussed below), including septin ring organization. Septin mutants form unstable septin rings, are multinucleate, and are defective for invasive growth (69). Rsr1^C244S^ expression was able to rescue two of these phenotypes but not agar penetration, implying that hyphal invasion is governed by a separable Rsr1-mediated pathway that requires stable association of Rsr1 with the PM and/or palmitoylation-dependent targeting to specific lipid domains. We only detected 2 phenotypes on deletion of the Rsr1 GEF, Bud5, and these were yeast specific, raising the possibility that Rsr1 is regulated via an alternative mechanism in hyphae. As Bud2 was required and its deletion mutant phenocopied that of Rsr1^G12V^, Bud2 appears to be the only GAP for Rsr1 in yeast and hyphae. Our pulldown assays did not identify Bud5 and Bud2 as Rsr1 interactors, but we were able to show this association by far-Western blot.

### Unpalmitoylated Rsr1^C244S^ rescues septin ring organization, vacuole fusion, and Spk stability.

Group 4 phenotypes—septin ring organization, vacuole fusion, and Spk stability—were restored when Rsr1 was able to undergo GDP-GTP cycling and to associate with membranes, as cells expressing GDP- or GDP-locked Rsr1 isoforms or cytoplasmic Rsr1^C245A^ exhibited the *rsr1*Δ null phenotype. This group was defined by Bud5 independence and rescue by expression of Rsr1^C244S^ (Fig. 10), supporting the idea that this farnesylated isoform underwent GDP-GTP cycling and was functional at vacuolar membranes to direct fusion. The rescue of septin ring assembly and Spk stability at the cell periphery further supports the idea that it Rsr1^C244S^ is trafficked to the PM, despite its lack of a secondary targeting motif, before recycling to endomembranes. Retrograde cycling of depalmitoylated Ras-family GTPases (Ras and Rap1) requires association with cytoplasmic chaperones (Vps35 [vacuolar protein sorting 35] and Cap1/Srv2, respectively), which sequester the hydrophobic acyl group (68, 70–73). The C. albicans genome contains homologs of Vps35 and Srv2, but the absence of the C-terminal CAAX box in our GST-6×His-Rsr1^1–143^ bait prohibited further investigation of this pathway. However, in S. cerevisiae, ∼10% of Rsr1 was cytosolic, presumably resulting from retrograde trafficking (36).

In S. cerevisiae, the roles of Cla4 in Cdc42-mediated septin ring assembly and vacuole tethering and docking are well characterized (16, 21, 22, 37, 62). Here, we showed that Cla4 or Rsr1 deletion in C. albicans produced similar vacuole fragmentation and mislocalized septin phenotypes. We also identified 5 vacuolar proteins and 5 septins in Rsr1 pulldown assays, strongly linking Rsr1 function to Cdc42-Cla4-mediated activity. The results also point to a distinction between phenotypes that are rescued through farnesyl-mediated Rsr1 association with the PM (yeast morphology, septin ring assembly, and Spk stability) and those that additionally require palmitoylation for more stable PM association and/or raft/domain targeting (bud site selection and substrate penetration) (Fig. 10). None of the phenotypes described above was rescued by expression of cytoplasmic Rsr1^C245A^.

### Rsr1-GDP rescues precocious nuclear division, apical dominance, and hyphal bursting.

The phenotypes in group 5 encompassed multinucleate cells and hyperbranched hyphae, which together indicated precocious cell cycle activation in cells distal to the hyphal tip and loss of apical dominance. Rescue was achieved by expression of either GDP-locked Rsr1^K16N^ or Rsr1^C245A^ (Fig. 10). As the Rsr1^C245A^ construct was cytosolic and failed to rescue 6 *rsr1*Δ phenotypes, it was initially thought to be nonfunctional. However, its ability to phenocopy *rsr1^K16N^* implied a functional relationship between the two isoforms that was likely to be their constitutive GDP-bound state; Rsr1^K16N^ through nucleotide bind-site mutation and Rsr1^C245A^ through its own intrinsic GTPase activity and no access to membrane-associated GEFs. The inability of Rsr1^C245A^ to completely rescue hyphal tip lysis, which likely occurred through disorganized cell wall delivery, suggests separable site-specific functions for Rsr1: cytosolic Rsr1-GDP was able to rescue cell cycle but not PM-associated phenotypes, while PM-associated Rsr1-GDP completely rescued both. Fungal Ras1 drives signaling from differential membrane platforms (74, 75), and our findings for Rsr1 support emerging examples of GTPases signaling from the cytosol. In D. melanogaster, eye development was supported by cytosolic Ras1 even though its other functions required CAAX box prenylation (67), and in C. albicans, cytosolic Ras1^C288S^ rescued specific cAMP-dependent growth and filamentation phenotypes (42). Taken together, there is mounting evidence that membrane association is not strictly required for some functions of Ras family GTPases.

How might expression of Rsr1-GDP rescue group 5 *rsr1*Δ phenotypes? In multinucleate cells, asynchronous nuclear division prior to septal closure indicated that the cell size checkpoint initiated START iteratively within the same body of cytoplasm. The observation that hyphal compartments had double septa is consistent with this, as START also signals the assembly of a new septin ring at the growing tip (76). Although aberrant nuclear inheritance contributed to the phenotype, precocious re-entry into START appeared to be the primary mechanism underlying the generation of multinucleate compartments and led to a 3-fold increase in the formation of new hyphal branches. In a phenotypic background where cytoplasm and mitochondria were retained subapically, Rsr1-GDP played a novel isoform-specific role in suppressing START in cells that are otherwise primed to transit the cell size checkpoint. Furthermore, this was independent of Rsr1-GDP localization. Rsr1-GDP cannot simply inhibit START, as its constitutive expression would be lethal. Instead, Rsr1-GDP may interfere with signaling at a specific stage of the cell cycle by interacting with, or buffering, drivers of START. This mechanism has been suggested for Rap1, the mammalian Rsr1 homolog, which suppresses oncogenic Ras by “diluting” Ras in signaling nanoclusters and obstructing interaction with its effectors, thereby dampening cell proliferation (77).

The observation that the *cla4*Δ mutant is also multinucleate (11) suggests Rsr1 signaling is epistatic to Cla4 activity. The paradigm is that Rsr1 lies upstream of Cdc42-Cla4 signaling, but studies in S. cerevisiae suggest Rsr1 also lies downstream of Cdc42-Cla4 from late G_1_ through mitosis, when Cla4 phosphorylates the GEF domain protein, Lte1. Cla4 and Lte1 localize at the daughter cell cortex, where Lte1 interacts with both Rsr1 and Ras to suppress activation (39, 40, 78). Our observation that PM-associated Rsr1-GDP was biased toward the hyphal apex is consistent with this. In S. cerevisiae, Cla4-Lte1 activity was viewed as a bifurcated signaling system that inhibits both inappropriate Rsr1-mediated polarization and Ras-mediated START. However, our observation that expression of Rsr1-GDP in a WT Ras1 background can suppress hyperactivation of START implies that Rsr1-GDP inhibits or interferes with Ras1 signaling, perhaps by direct binding, as Ras1 was pulled down in our Rsr1 interactor assays (Fig. S2). A further player in this interaction may be Bem1, which specifically binds Rsr1-GDP during G_1_ in S. cerevisiae (35, 36). However, whether Bem1 acts as a scaffold for Cla4-Lte1-mediated Rsr1-Ras1 inhibition or is sequestered by Rsr1-GDP to avoid inappropriate polarity complex formation remains to be determined.

## MATERIALS AND METHODS

### Growth conditions and media.

The C. albicans strains used in this study are listed in Table 2. Yeast was grown in YPD medium (1% [wt/vol] yeast extract [Oxoid], 2% [wt/vol] mycological peptone [Oxoid], 2% [wt/vol] glucose) at 30°C. Uridine prototrophs were selected on synthetic defined (SD) medium (0.67% [wt/vol] yeast nitrogen base [YNB; Difco], 2% [wt/vol] glucose). Hyphae were induced in 20% (vol/vol) fetal bovine serum (FBS; Sigma) with 2% glucose (wt/vol) at 37°C. E. coli strains were grown in Luria-Bertani (LB) medium (1% [wt/vol] tryptone, 0.5% [wt/vol] yeast extract, 0.5% [wt/vol] NaCl) at 37°C.

**TABLE 2:** C. albicans strains used in this study

Strain	Details	Reference or source
BWP17	*ura3*::λ*imm434/ura3*::_λ *imm434 his1*::*hisG/his1*::*hisG arg4*::*hisG/arg4*::*hisG*	88
DAY185	*ura3*::*imm434/ura3*::*imm434 his1*::*hisG*::*HIS1/his1*::*hisG arg4*::*hisG*::*ARG4-URA3/arg4*::*hisG*	89
JB6284/9955	(BWP17) *his1*::*hisG/hisG*::*his1*::*HIS1 arg4*::*hisG/ARG4-URA3*::*arg4*::*hisG*	90
CA8832	(BWP17) *rsr1*::*ARG4*/*rsr1*::*HIS1*	5
CA8880	(BWP17) *rsr1*::*ARG4/rsr1*::*HIS1 arg4*::*hisG/ARG4-URA3*::*arg4*::*hisG*	5
MG9215	(BWP17) *rsr1*::*ARG4*/p*URA3*-*RSR1*::*rsr1*::*HIS1*	5
DH7453	(BWP17) *bud2*::*ARG4/bud2*::*HIS1 arg4*::*hisG/ARG4-URA3*::*arg4*::*hisG*	5
CA8855	(BWP17) *bud5*::*HIS1*/*bud5*::*ARG4 arg4*::*URA3-ARG4*	C. Gale, unpublished
CA10055	(BWP17) *URA3*-P*_MET3_*-*YFP*::*BUD2/BUD2*	32
CA12343	(BWP17) *URA3-*P*_MET3_-YFP*::*BUD5/BUD5*	32
pACT1-GFP	CAI-4 (*ura3*::*λimm434/ura3*::*λ imm434*) *RPS1/RPS1*::*pACT1-GFP*	91
A498	(CA8832) *rsr1*::*ARG4*/*pURA3*-*rsr1*^G12V^::*rsr1*::*HIS1*	This study
A500	(CA8832) *rsr1*::*ARG4*/*pURA3*-*rsr1^K16N^*::*rsr1*::*HIS1*	This study
A494	(CA8832) *rsr1*::*ARG4*/*pURA3*-*rsr1^C244S^*::*rsr1*::*HIS1*	This study
A582	(CA8832) *rsr1*/*rsr1 RPS1*::*RPS1-URA3-ACT1p-YFP-RSR1-CYCt*	This study
A572	(CA8832) *rsr1*/*rsr1 RPS1*::*RPS1-URA3-ACT1p-YFP-rsr1^G12V^-CYCt*	This study
A574	(CA8832) *rsr1*/*rsr1 RPS1*::*RPS1-URA3-ACT1p-YFP-rsr1^K16N^-CYCt*	This study
CA8870	*bud2*::*HIS1/bud2*::*ARG4 ura3Δ*::*λimm434/ura3Δ*::*imm434 his1*::*hisG/his1*::*hisG arg4*::*hisG/arg4*::*hisG MLC1/MLC1*::*YFP-URA3*	C. Gale, unpublished
A577	(CA8832) *rsr1*/*rsr1 RPS1*::*RPS1-URA3-ACT1p-YFP-rsr1^C244S^-CYCt*	This study
A578	(CA8832) *rsr1*/*rsr1 RPS1*::*RPS1-URA3-ACT1p-YFP-rsr1^C245A^-CYCt*	This study
YJB8860	(BWP17) *NOP1/NOP1-YFP:URA3 CDC3/CDC3-GFP*::*HIS1*	12
A827	(YJB8860) *rsr1*/*rsr1 eno1*Δ::CaCas9-FRT	This study
A786	(DAY185) *CDC11*::*CDC11-GFP-ADH1t-NAT1*	This study
A789	(CA8880) *rsr1*Δ/Δ *CDC11*::*CDC11-GFP-ADH1t-NAT1*	This study
BWP17-mtGFP	(BWP17) *RP10*::*RP10-URA3-pACT1-mtGFP-CYCt*	64
A762	(CA8832) *rsr1*/*rsr1 RP10*::*RP10-URA3-pACT1-mtGFP-CYCt*	This study
A908	(DAY185) *DBF2*::*DBF2-GFP-ADH1t-NAT1*	This study
A902	(CA8880) *rsr1*/*rsr1 DBF2*::*DBF2-GFP-ADH1t-NAT1*	This study
MG7139	(BWP17) *MLC1/MLC1-YFP-URA3*	26
CA9151	(BWP17) *rsr1*::*ARG4/rsr1*::*HIS1 MLC1/MLC1-YFP-URA3*	32
A940	(CA8855) *bud5*/*bud5 MLC1/MLC1*::*YFP-NAT1*	This study
A941	(A498) *rsr1/rsr1^G12V^ MLC1/MLC1*::*YFP-NAT1*	This study
A944	(A500) *rsr1/rsr1^K16N^ MLC1/MLC1*::*YFP-NAT1*	This study
A948	(A494) *rsr1/rsr1^C244S^ MLC1/MLC1*::*YFP-NAT1*	This study
A950	(A578) *YFP-rsr1^C245A^ MLC1/MLC1*::*YFP-NAT1*	This study
CLJ5	*ura3/ura3 cacla4*Δ::*hisG*/Ca*cla4*Δ::*hisG*	11

### C. albicans strain construction.

Plasmids and primers are listed in Tables 3 and 4, respectively.

**TABLE 3:** Plasmids used in this study

Plasmid	Details	Reference
pMG2128	*pGEM-URA3-RSR1*	5
pAB146	*pMG2128 rsr1^G12V^*	This study
pAB147	*pMG2128 rsr1^K16N^*	This study
pAB144	*pMG2128 rsr1^C244S^*	This study
pACT1	*pURA3-ACT1p-CYCt*	80
pAB158	*pACT1-YFP-RSR1-CYCt*	This study
pAB204	*pACT1-YFP-rsr1^G12V^-CYCt*	This study
pAB209	*pACT1-YFP-rsr1^K16N^-CYCt*	This study
pAB210	*pACT1-YFP-rsr1^C244S^-CYCt*	This study
pAB213	*pACT1-YFP-rsr1^C245A^-CYCt*	This study
pET41b_FT	*T7p-GST*-*6His*-PreScission*-8His-T7t*	83
pAB280	*T7p-GST-6His-Rsr1^1-243^-8His-T7t*	This study
pAB285	*T7p-GST-6His*-*8His-T7t*	This study
mtGFP	*pACT1*-mtGFP	64

**TABLE 4:** Primers used in this study

No.	Primer name	Sequence (5′→3′)
1	RSR1-C244S-F^*a*^	GTCTAGCTCAGGATCCAAG**AGC**TGCAC
2	RSR1-C244S-R	GTGCAGCTCTTGGATCCTGAGCTAGAC
3	RSR1-K16N-F^*b*^	GTATTGGGCGCCGGTGGGGTAGGT**AAT**TCCTCAATCACCG
4	RSR1-K16N-R	CGGTGATTGAGGAATTACCTACCCCACCGGCGCCCAATAC
5	RSR1-G12V-F^*c*^	CGTAGTACTTGGTGCT**GTT**GGGGTAGG
6	RSR1-G12V-R	CCTACCCCAACAGCACCAAGTACTACG
7	YR-G12V-F^*d*^	CGTAGTATTGGGTGCT**GTT**GGGGTAGGTAAATCCT
8	YR-G12V-R	AGGATTTACCTACCCCAACAGCACCCAATACTACG
9	YR-K16N-F^*e*^	GTGCTGGTGGGGTAGGT**AAT**TCCTCAATCACC
10	YR-K16N-R	GGTGATTGAGGAATTACCTACCCCACCAGCAC
11	YR-C244S-F^*f*^	CTAGCTCAGGAAGCAAG**AGC**TGCACAATTATGTGA
12	YR-C244S-R	TCACATAATTGTGCAGCTCTTGCTTCCTGAGCTAG
13	YR-C245A-F^*g*^	TAGCTCAGGAAGCAAGTGC**GCC**ACAATTATGTGAGTCGAC
14	YR-C245A-R	GTCGACTCACATAATTGTGGCGCACTTGCTTCCTGAGCTA
15	p41_GST-F^*h*^	GGAAGTTCTGTTCCAGGGGCCCGATATCTCTACA
16	p41_GST-R	GGTGGTGGTGGTGGTGGTGCTCGAGCTATAGTGTCGT
17	41_GR-F	CACACAGGGCCCAGAGATTATAAAGTCGTAGTATTGGGTGC (anneals after *RSR1* intron)
18	41_GR^1-243^-R	GGTTGGCTCGAGTTACTTGCTTCCTGAGCTAGACTTTGGGTG
19	Dbf2-GFP-F^i^	acaatggtgaaataaacttattgaatatggtcgaaaatggaaatggaattggaaatggaaattctcgatcaagtagattaaatccattagctacattgtatGGTGGTGGTTCTAAAGGTGAAGAATTATT
20	Dbf2-GFP-R^*i*^	aaactaaatcaagccaaatctctacgagtttacaattctaatatagttttctaatcattatacaacatcctaaattaatcaaacaaacaccatttaatataCGTTAGTATCGAATCGACAGC
21	Cdc11-GFP-F^*i*^	ggaaatcgaaaagagattgttggccgaagggttaaagtttgatgaaaatggtgatgtagttaaagtacacgaagaggagtcttcagaaaatgaagtaaaagtaatcGGTGGTTCTGGTGGTGGTTCTGGTGGTGGTTCTAAAGGTGAAGAATTATT
22	Cdc11-GFP-R^*i*^	cagagttgttgagcgagttgaaacgattggacgcttcacacgagttgttgaaagagcaagatctcaaaacaagtatatttgacgacattgttacaaaatattctatCGTTAGTATCGAATCGACAGC
23	Mlc1-YFP-F^*i*^	ggtgaaaagttgactgactctgaagttgatgagttattaaaaggggtcaatgtaacttctgatggaaatgtggattatgttgaatttgtcaaatcaattttagaccaaGGTGGTGGTTCTAAAGGTGAAGAATTATT
24	Mlc1-YFP-R^*i*^	cttcaaataaacggtatccaattcgaacaagactatacaataactataatttgtaaaacttgtagtatatatatttcaatggttaattgtaaattttcttttatCGTTAGTATCGAATCGACAGC
25	G142-F	ATTTGAATCACCGTGCAATTTGTCCG
26	G142-R	AAAACGGACAAATTGCACGGTGATTC
27	R142-F^*j*^	GTATTGGGTGCTGGTGGGGTAGGTAAATCCTCAATCACCGTGCAATTTGTCC**TAA**GATATCTGTA
28	R142-R^*j*^	TATAGGAGTCTTCAATTGTAGGGTCGTAACTTTCGACGTATACAGATATC**TTA**GGACAAA

a BamHI cut site is underlined, C244S mutation is in bold.

b NarI cut site is underlined, K16N mutation is in bold.

c ScaI cut site is underlined, G12V mutation is in bold.

d G12V mutation is in bold.

e K16N mutation is in bold.

f C244S mutation is in bold.

g C245A mutation is in bold.

h EcoRV cut site is underlined.

i Lowercase letters indicate allele homology, uppercase letters indicate FP-NAT1 cassette homology, and serine-glycine linker is underlined.

j EcoRV cut site is underlined, stop codon is in bold.

**(i) Site-directed mutagenesis of *RSR1*.** Vector pMG2128 (5), containing *RSR1* open reading frame (ORF) CR_02140W_A 825 bp upstream of the promoter and 240 bp downstream of the stop codon, was modified by site-directed mutagenesis (SDM) using primer pairs 1-2, 3-4, and 5-6, complementary to the *RSR1* ORF. Primer silent mutations introduced BamHI (C244S), NarI (K16N), and ScaI (G12V) cut sites for screening. Resulting plasmids pAB146 (Rsr1^G12V^), pAB147 (Rsr1^K16N^), and pAB144 (Rsr1^C244S^) were sequenced before linearization with MluI and integrated into the promoter of the disrupted *rsr1* locus in CA8832 (*rsr1*Δ/*rsr1*Δ, Ura^−^), generating strains A498, A500, and A494. Cells were selected for uridine prototrophy and confirmed by PCR.

**(ii) FP-tagging and SDM of YFP-*RSR1*.** The codon-optimized YFP sequence with a Start codon (79) was integrated upstream of the *RSR1* ORF (CR_02140W_A including the intron between nucleotides [nt] 8 to 81, inclusive) with a 6-amino-acid (aa) linker (CCGCCATGGCTCGAGGGT). The fragment encoding YFP-RSR1 and flanked by MluI (5′) and SalI (3′) sites was synthesized by GeneArt (Life Technologies) and inserted between MluI and SalI sites in the pACT1 plasmid (80) to create pAB158. Plasmid pAB158 was used as a template for SDM using primer pairs 7-8, 9-10, 11-12, and 13-14, complementary to the *RSR1* ORF, resulting in plasmids pAB204 (YFP-Rsr1^G12V^), pAB209 (YFP-Rsr1^K16N^), pAB210 (YFP-Rsr1^C244S^), and pAB213 (YFP-Rsr1^C245A^), respectively. Plasmid DNA was linearized with StuI and integrated into the *RPS1* locus. Transformants were confirmed by PCR, generating strains A582, A572, A574, A577, and A578. Plasmid pACT1-mtGFP bearing a mitochondrial localization signal (64) was linearized by StuI and integrated at the *RPS1* locus of CA8832. Cells were selected and integration was confirmed as described above.

**(iii) PCR-mediated C-terminal FP tagging: Cdc11-GFP, Mlc1-YFP, and Dbf2-GFP.** Cdc11 (C5_00070W_A), Mlc1 (CR_03090C_A), and Dbf2 (C2_06670C_A) were C-terminally tagged by amplifying the FP-nourseothricin (NAT) cassette from plasmids pGFP-NAT1 or pYFP-NAT1 (81) using primer pairs 21-22, 23-24, and 19-20, respectively. Cells were selected on YPD agar containing 300 μg/ml nourseothricin (Sigma), and integration of the GFP-NAT cassette was confirmed by PCR.

**(iv) CRISPR-Cas9 to disrupt *RSR1* ORF in Nop1-YFP Cdc3-GFP background.** The CRISPR-Cas9 system for C. albicans (82) was used to introduce a frameshift, cut site, and a stop codon in the *RSR1* ORF (CR_02140W_A). A unique guide sequence (AATCACCGTGCAATTTGTCC) targeting both alleles 142 nt from the *RSR1* start codon (located outside the intron from 8 to 81 nt) was cloned into plasmid pV1200. A mutagenic repair DNA template (primer pair 27-28) containing a stop codon, a cut site, and a frameshift was amplified, and pV1200 containing *RSR1* PAM-142 (primer pair 25-26) was cut with KpnI and SacI and cotransformed into 8860 (Nop1-YFP Cdc3-GFP), generating strain A827 (*rsr1*Δ/Δ Nop1-YFP Cdc3-GFP). Cells were selected on YPD agar with 300 μg/ml nourseothricin. The NAT resistance marker was recycled, and the *RSR1* ORF was sequenced.

**(v) Heterologous expression of GST-Rsr1^1–243^.** *RSR1* was amplified from plasmid pAB158 using primer 17 (containing the first two *RSR1* codons and annealing to *RSR1* after the intron) and 18 (annealing upstream of 243-Cys), digested with ApaI and XhoI, and ligated into pET41b-FT, a derivate of commercial plasmid pET41b (Invitrogen) (83), to generate pAB280 (Table 3). A GST-only control was generated by amplifying pET41b-FT with primer pair 15-16 to introduce an EcoRV cut site on each side of the fragment. EcoRV digestion followed by ligation generated plasmid pAB285. Constructs were sequenced and transformed into E. coli BL21(DE3).

### Fluorescence microscopy and image analysis.

**(i) Mlc1-YFP imaging in PDMS chambers.** Polydimethylsiloxane (PDMS) (Sylgard-184; Dow-Corning) chambers were used for imaging Mlc1-YFP as described by Thomson et al. (8). Cells were diluted in 100 μl 20% FBS with 2% glucose and spread on 1.5-cm^2^ PDMS attached to a slide. Images were captured every 3 min on an inverted Zeiss AxioObserver Z1 using a Plan-Neofluor 40×/1.3 numerical aperture (NA) oil immersion lens objective (Carl Zeiss) and a 16-bit CoolSNAP H2 charge-coupled-device (CCD) camera (Photometrics) with attached incubation chamber (PeCon GmbH) at 37°C. YFP was imaged using a YFP-2427B-ZHE-ZERO filter (Laser 2000). Spk positioning of Mlc1-YFP was plotted in hyphae for (i) nonlinear sequential positioning of Mlc1-YFP and (ii) loss of ridge contact. Septal closure duration was determined where *t* = 0 represents the time of appearance of Mlc1-YFP at the septum (26).

**(ii) YFP-Rsr1, YFP-Rsr1^G12V^, and YFP-Rsr1^K16N^ fluorescence intensity analysis.** Hyphae were grown on a poly-l-lysine slide for 5 h. Images were acquired as described, with 2 × 2 binning and a 3-s exposure. YFP was imaged as before. Images were deconvolved in AxioVision (Rel. 4.8) software, and YFP intensity was measured at the plasma membrane along the 10-μm region subapical to the hyphal tip using ImageJ (v. 1.45s).

**(iii) FM4-64 staining and analysis of vacuolar area.** Vacuoles were stained with the lipophilic styryl red dye FM4-64 (Molecular Probes, Life Technologies). Hyphae were grown on a poly-l-lysine slide for 5 h and stained with 16 μM FM4-64 for 45 min at 37°C in the dark, washed with double-distilled water (ddH_2_O), and incubated in fresh 20% FBS for 1 h at 37°C. Samples were co-stained with 5 μg/ml calcofluor white (CFW) (Sigma), and 0.4-μm optical sections were captured on an UltraVIEW VoX spinning disk confocal microscope (Nikon, Surrey, UK) using 561-, 514-, and 405-nm lasers and running Volocity 6.3 software (PerkinElmer). Vacuolar area (μm^2^) was determined by tracing vacuoles in a two-dimensional (2D) format of a maximum projection of optical sections using ImageJ (v. 1.52p). Total cell volume was measured by tracing CFW-stained nonbranched subapical compartments. The proportion of cytoplasm per hyphal compartment was calculated by subtracting vacuolar area from total cell area.

**(iv) Live-cell imaging of FP-tagged proteins.** Hyphae were grown in an 8-well ibidi μ-slide (Ibidi) and imaged every 3 min, or as indicated, on a spinning disk confocal microscope. Optical sections were acquired, and images are maximum intensity projections, unless otherwise stated. Where indicated, the growth medium was supplemented with 2 μg/ml CFW. For mitoGFP, hyphae were incubated for 5 h at 37°C and stained with 5 μg/ml CFW. Optical sections at 0.4-μm increments were captured on a spinning disk confocal microscope, using 488-and 405-nm lasers. The mean fluorescence intensity was analyzed for 5-μm^2^ areas in the middle of the nonbranched subapical hyphal compartments in ImageJ (v. 1.52p).

**(v) Quantification of nuclei and cell diameter.** Hyphae were grown on a poly-l-lysine slide for 5 h and fixed in 4% formaldehyde. Nuclei were stained with 1.2 mM DAPI (Sigma) or with Vectashield with 1.5 mg/ml DAPI (Vector Laboratories). Hyphae were co-stained with 0.1 μg/ml CFW where indicated. Optical sections were captured on the Zeiss Axio Observer (40×/1.3 NA), using a DAPI filter. Nondividing yeast cells were analyzed for nuclear number and cell diameter.

**(vi) CFW staining of bud scars.** Hyphae were grown as before and stained with 10 μg/ml CFW. Bud scars were visualized after 16 h of incubation at 30°C. Yeast cells were washed with ddH_2_O and stained with 15 μg/ml CFW. Z-stacks were captured on a Zeiss LSM880 with Airyscan running Zen 2.3 SP1, with maximum intensity projections generated in Zen.

**(vii) Cell lysis and secondary growth axis analysis** Cell lysis was determined with live imaging of cells incubated in either YPD at 30°C or 20% FBS with 2% glucose and with or without 1 M sorbitol at 37°C in an 8-well ibidi μ-slide (ibidi). Images were acquired every 5 min using a differential interference contrast (DIC) filter. Cell lysis was quantified for each frame until no new cell death was detected (≈1 h). The total number of axes per primary hypha was determined after 5 h of incubation.

### Heterologous expression and purification of recombinant GST-Rsr1 proteins.

Overnight E. coli BL21(DE3) bacterial cultures were diluted to an optical density at 600 nm (OD_600_) of 0.1 in 1 L LB broth supplemented with 25 μg/ml kanamycin and grown to an OD_600_ of 0.8. Expression was induced in 1 mM IPTG (isopropyl β-d-1-thiogalactopyranoside), and cultures were incubated overnight at 30°C, 200 rpm. Cells were lysed with low-salt buffer (20 mM Tris-HCl, 50 mM NaCl, 1 mM phenylmethylsulfonyl fluoride [PMSF], pH 7.4) and freezing, followed by incubation with 80 mg lysozyme for 1 h at 4°C and sonication. Cell lysate were ultracentrifuged (45,000 × *g*, 45 min, 4°C), and the supernatant was retained. Protein extracts containing GST-Rsr1 were applied to glutathione columns (GSTrap 4B columns; GE Healthcare Life Sciences) previously equilibrated in GST buffer (20 mM Tris, 150 mM NaCl, pH 7.4). Columns were washed with 20 mM Tris and 500 mM NaCl (pH 7.4), and proteins were eluted with GST basis buffer containing 20 mM glutathione.

### Affinity purification (pulldown) and mass spectrometry of GST-Rsr1 with C. albicans lysate *in vitro*.

After growth from an OD_600_ of 0.05 to an OD_600_ of 0.8 in YPD at 30°C and 200 rpm, for hyphae, overnight cultures were diluted to an OD_600_ 0.05 in 20% FBS and incubated at 37°C and 200 rpm to an OD_600_ of 0.35. Cells were lysed with acid-washed glass beads in lysis buffer (50 mM Tris [pH 8], 150 mM NaCl, 0.5% NP-40, 2 μg/ml leupeptin, 2 μg/ml pepstatin, 1 mM PMSF). Purified recombinant GST-Rsr1 (550 μg) was combined with 45 mg crude protein extract from C. albicans yeast or hyphae. Samples were incubated overnight on a rotor at 4°C. Glutathione Sepharose beads (500 μl) were equilibrated in phosphate-buffered saline (PBS), added to protein mixtures, and rotated for 3 h at 4°C. Beads were pelleted and washed with PBS. Proteins were eluted in 100 μl PBS with 20 mM glutathione and digested for 8 h at 37°C on an Investigator ProGest robotic workstation (Digilab Ltd., Huntingdon, UK). Peptide solutions were dried in a concentrator (SPD1010 Savant SpeedVac; Thermo Fisher Scientific), dissolved in 10 μl 0.1% formic acid, and analyzed by liquid chromatography tandem mass spectrometry (LC-MS/MS) using an QExactive Orbitrap mass spectrometer (Thermo Fisher Scientific). Mass lists were input to Mascot MS/MS ion searches (version 2.2; Matrix Science, London, UK) against sequence files from *Candida* Genome Database. Area values were calculated from the ion intensities of unique peptides identified for each protein. Extracted ion chromatograms for the 3 most abundant peptides were reconstructed in Mascot, and the area under the peak was calculated. Area values of proteins copurified with Rsr1 were normalized per sample using a ratio over the sum area value. Normalized values of individual proteins were divided by their normalized values in the negative control (nonspecific binding to the GST tag).

### Far-Western blotting.

The protocol was according to that described by Wu at al. (84). Briefly, crude protein lysates (100 μg) containing YFP-tagged “prey” proteins were loaded onto an SDS (bis-Tris) polyacrylamide (4% to 12%) gel and transferred to a polyvinylidene difluoride (PVDF) membrane followed by denaturing and renaturing steps. The membrane was blocked with 5% milk and incubated with 5 μg (1 μg/ml) purified recombinant GST-Rsr1 “bait” protein in PBS with 5 mM MgCl_2_ for 3 h at room temperature (RT). GST-Rsr1 was detected with horseradish peroxidase (HRP)-conjugated anti-GST antibody (8-326; Thermo Fisher Scientific) using a chemiluminescence two-component kit (Pierce ECL Western blotting substrate; Thermo Fischer Scientific). The membrane was stripped using stripping buffer (Thermo Fisher Scientific) for 15 min at RT, and YFP-labeled proteins were detected by anti-GFP antibody (7.1 and 13.1; Roche), followed by HRP-conjugated anti-mouse antibody (7076; Cell Signaling Technology).

### Rsr1 3D structure prediction.

A homology model of Rsr1 (ORF CR_02140W_A) was generated using Yasara software (YASARA Structure 11.12.31) applying the following parameters: PsiBLASTs-1, EValue Max-0.5, Templates Total-5, Templates SameSeq-1, OligoState-4, Alignments-5, LoopSamples-50, TermExtensions-90, Speed-Slow. The core domain was modeled with a structural template identified by BLAST search in the Protein Data Bank (PDB) for a match, with a modeling E value cutoff of 0.5. Since the C terminus of Rsr1 is unique and does not show any homology to known protein structures, it was modeled by the YASARA algorithm for termini building.

### High-pressure freezing and transmission electron microscopy.

Cells were fixed by high-pressure freezing with an EMPACT2 high-pressure freezer and rapid transport system (Leica Microsystems Ltd.). Cells were freeze-substituted in substitution reagent (1% [wt/vol] OsO_4_ in acetone) with a Leica EMAFS2 and embedded in Spurr resin (Sigma). Additional infiltration was provided under a vacuum at 60°C before embedding in Leica FSP specimen containers and polymerized at 60°C for 48 h. Sections (60 nm) were prepared with a Diatome diamond knife on a Leica UC6 ultramicrotome and stained with uranyl acetate and lead citrate for examination with a JEOL 1400 plus transmission microscope (JEOL UK Ltd., Hertfordshire, United Kingdom) and imaging with an AMT UltraVUE camera (Deben).

### Agar invasion assay.

Cells were diluted to 2.5 × 10^4^ cells/5-μl spot on Spider agar (85). Colony diameter was imaged after incubation at 37°C for 6 days. Six colonies per strain were cross-sectioned and imaged against a ruler under a dissection microscope. Penetration depth was measured using a calibration tool and Openlab software (Agilent Technologies, Santa Clara, CA).

### Statistical analyses.

Data generated in this study were imported into GraphPad Prism V8 for Windows (GraphPad Software) or SPSS V26. The statistical tests used, level of significance, and error bars are described in figure legends.
