# Supplementary figures and images for: Rsr1 Palmitoylation and GTPase Activity Status Differentially Coordinate Nuclear, Septin, and Vacuole Dynamics in Candida albicans
Source: mBio. 2020 Oct 13;11(5):e01666-20. doi: 10.1128/mBio.01666-20 (PMC7554666; doi:10.1128/mBio.01666-20)

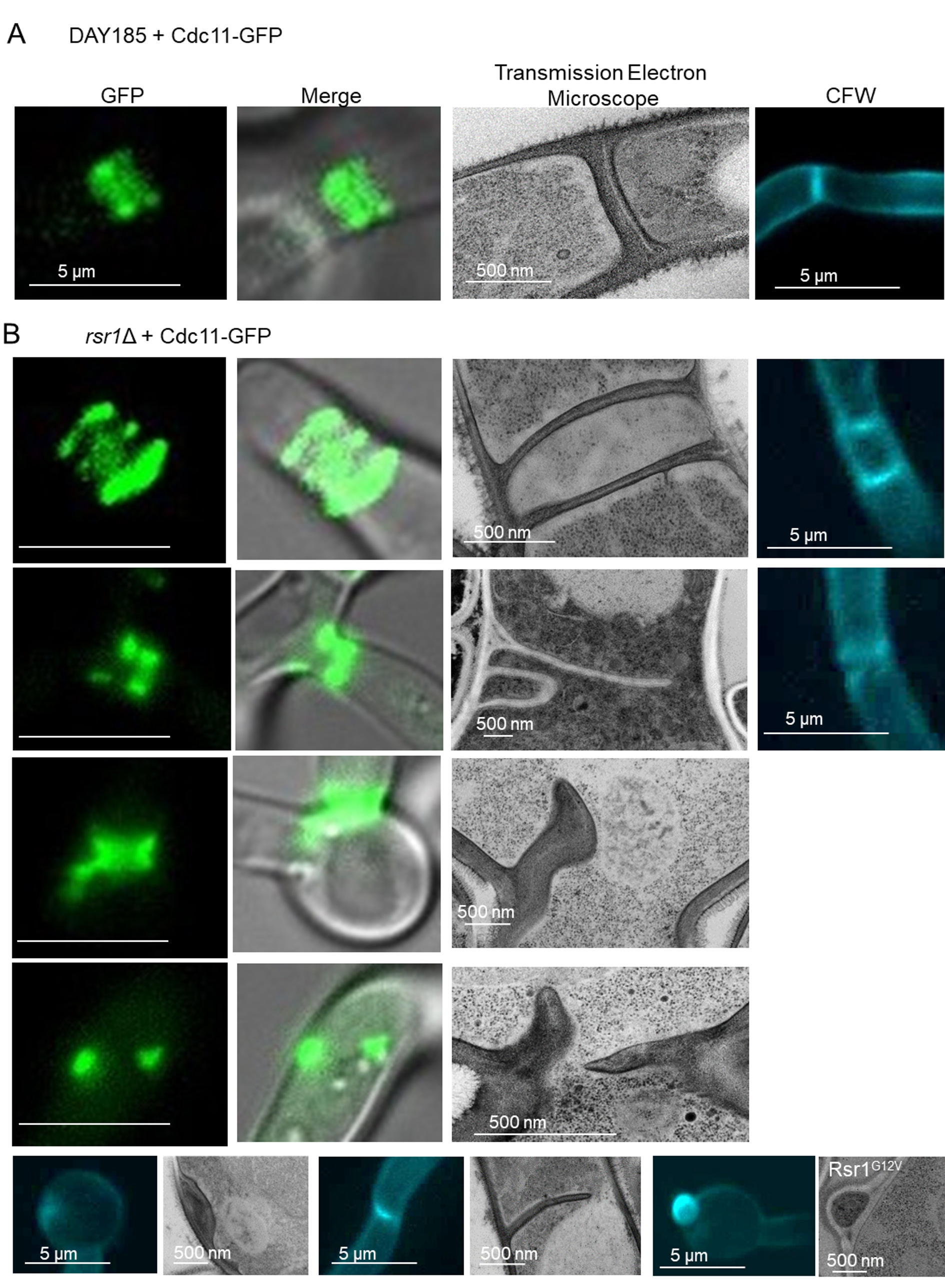

Supplement: FIG S1 [file mBio.01666-20-sf001.tif]

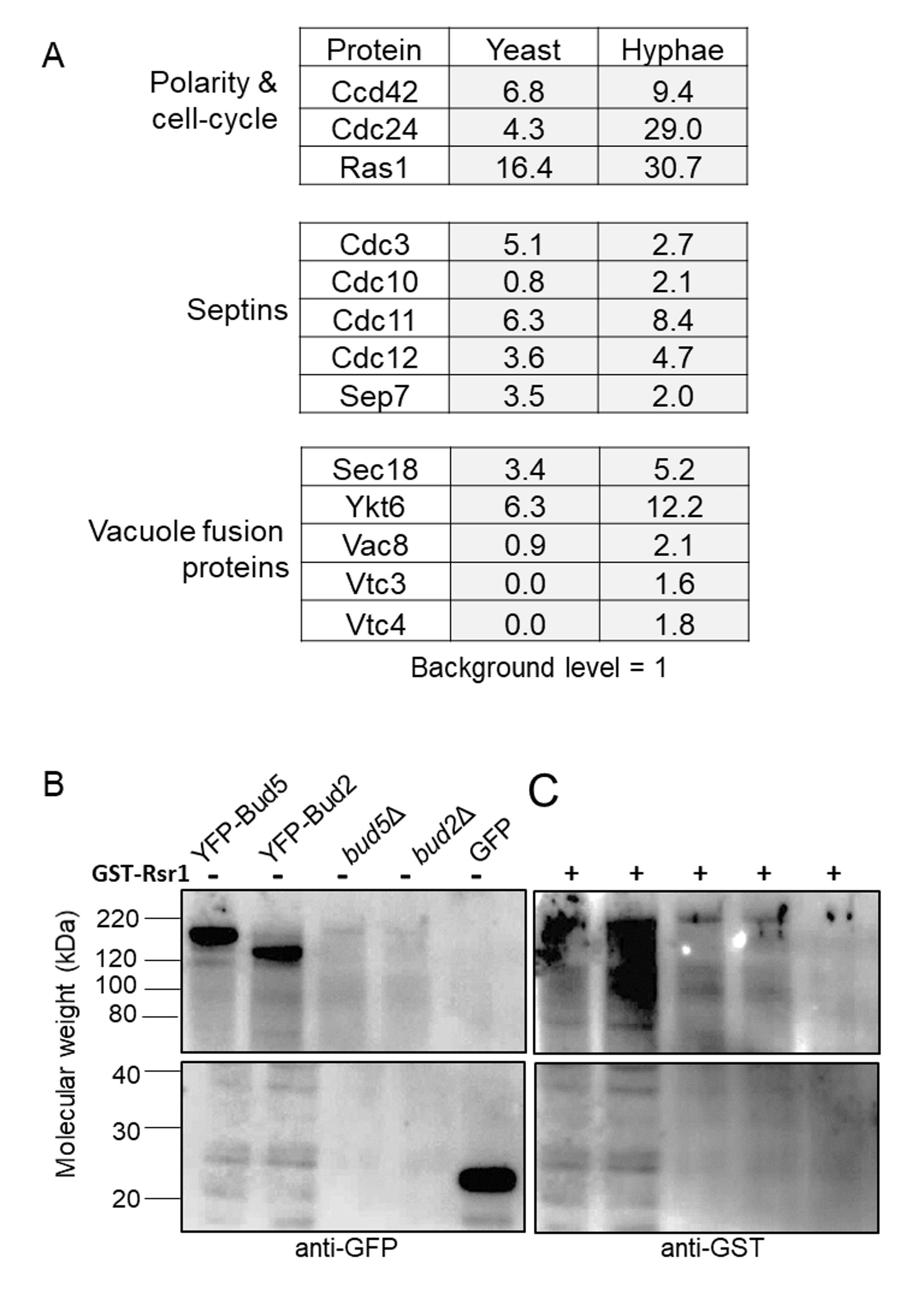

Supplement: FIG S2 [file mBio.01666-20-sf002.tif]

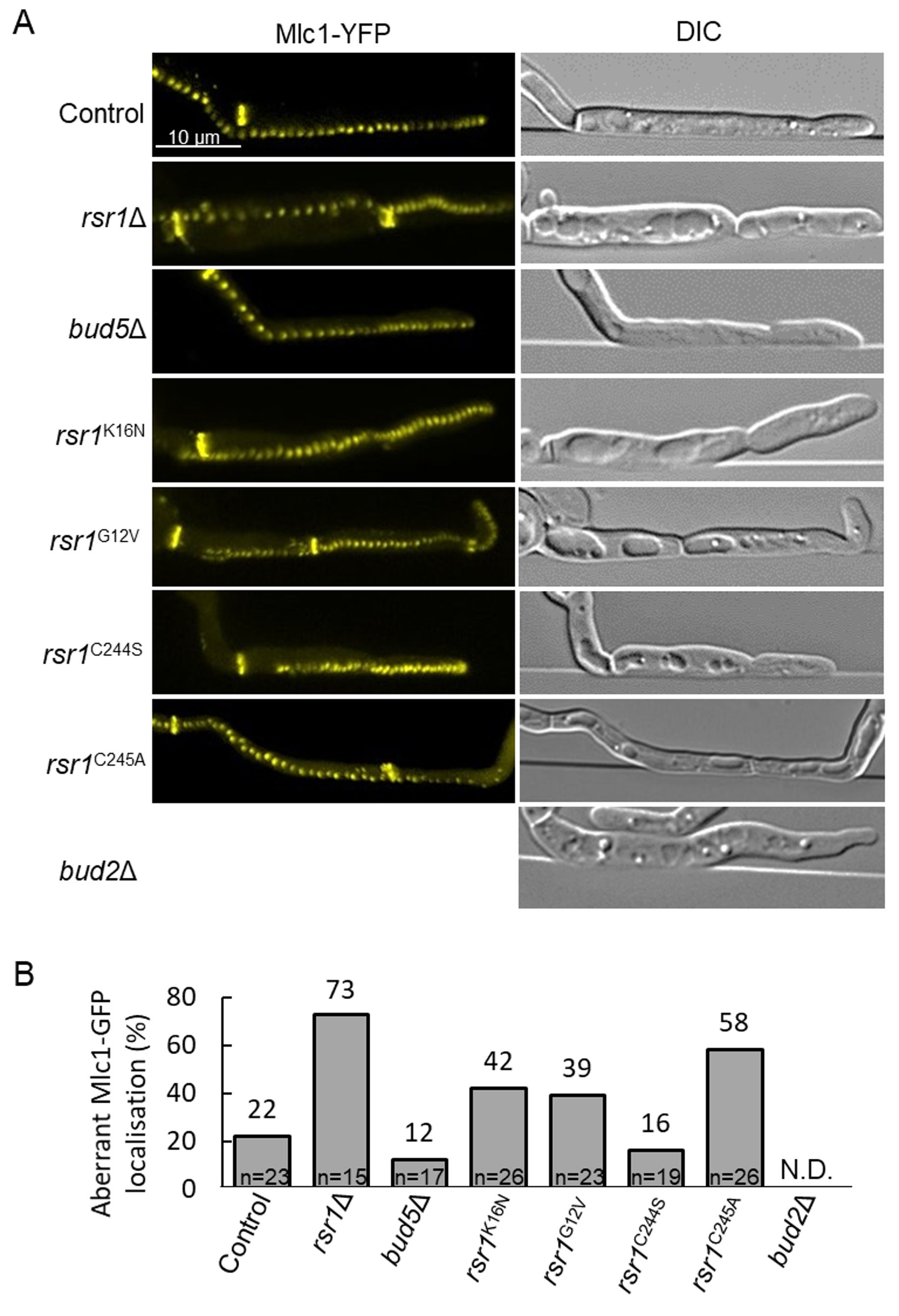

Supplement: FIG S3 [file mBio.01666-20-sf003.tif]

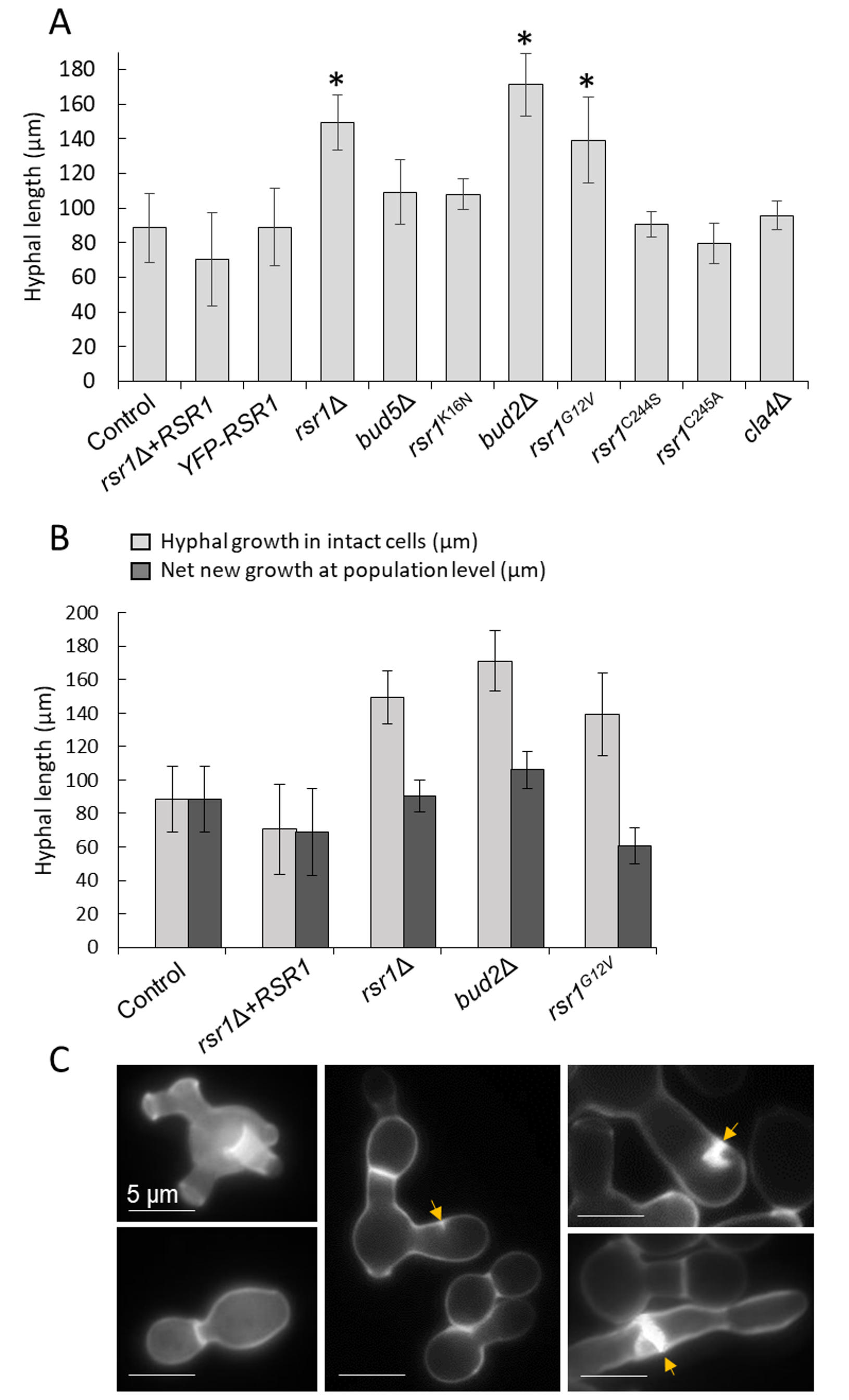

Supplement: FIG S4 [file mBio.01666-20-sf004.tif]
